# Incidental exposure to hedonic and healthy food features affects food preferences one day later

**DOI:** 10.1186/s41235-021-00338-6

**Published:** 2021-12-11

**Authors:** Léo Dutriaux, Esther K. Papies, Jennifer Fallon, Leonel Garcia-Marques, Lawrence W. Barsalou

**Affiliations:** 1 School of Psychology and Neuroscience, University of Glasgow, 62 Hillhead Street, Glasgow, G12 8QB UK; 2 CICPsi Research Center for Psychological Science, University of Lisbon, Lisbon, Portugal; 3 School of Psychology, University of Lisbon, Lisbon, Portugal; 4 Present Address: Center for Mind/Brain Science, University of Trento, Mattarello, Italy

**Keywords:** Eating, Food preference, Food exposure, Habits, Incidental learning, Indirect memory

## Abstract

Memories acquired incidentally from exposure to food information in the environment may often become active to later affect food preferences. Because conscious use of these memories is not requested or required, these incidental learning effects constitute a form of indirect memory. In an experiment using a novel food preference paradigm *(n* = 617), we found that brief incidental exposure to hedonic versus healthy food features indirectly affected food preferences a day later, explaining approximately 10% of the variance in preferences for tasty versus healthy foods. It follows that brief incidental exposure to food information can affect food preferences indirectly for at least a day. When hedonic and health exposure were each compared to a no-exposure baseline, a general effect of hedonic exposure emerged across individuals, whereas health exposure only affected food preferences for high-BMI individuals. This pattern suggests that focusing attention on hedonic food features engages common affective processes across the general population, whereas focusing attention on healthy food features engages eating restraint goals associated with high BMI. Additionally, incidental exposure to food features primarily changed preferences for infrequently consumed foods, having less impact on habitually consumed foods. These findings offer insight into how hedonic information in the obesogenic food environment contributes to unhealthy eating behavior that leads to overweight and obesity. These findings further motivate the development of interventions that counteract the effects of exposure to hedonic food information and that broaden the effects of exposure to healthy food information.

**Supplementary Information:**

The online version contains supplementary material available at 10.1186/s41235-021-00338-6.

## Significance

We demonstrate that incidentally acquired memories of hedonic and healthy food features influence eating preferences. We further demonstrate that even brief exposure to food information can have lasting effects for at least a day. Finally, we demonstrate that exposure to hedonic information generally affects most individuals, whereas exposure to healthy information primarily affects individuals high in BMI. These findings have significant implications for understanding eating cognition and behavior. They also have significance for developing interventions that discourage unhealthy eating and promote healthy eating. To establish these findings, we developed a novel experimental paradigm informed by basic research in cognitive psychology. Specifically, we examined how the classic memory processes of incidental learning and indirect memory combine to influence food preferences. We further demonstrated how individual differences can be integrated into this paradigm (BMI, healthy eating habits), along with differences in foods (tasty foods vs. healthy foods; habitually consumed foods vs. occasionally consumed foods). Finally, we developed an approach to mixed-effect modeling that focuses on establishing effect sizes and on assessing the generalizability of effects across participants and foods.

People are constantly exposed to diverse sources of food information that highlight various outcomes of food consumption, such as immediate hedonic pleasure, long-term health, and physical attractiveness. On the one hand, the food industry uses images and language to promote how tasty, filling, satisfying, and enjoyable consuming a food will be. On the other, health experts recommend reducing the consumption of foods high in fat, salt, and sugar, while increasing the consumption of foods that lead to health and longevity.

Previous research has established that exposure to food information in advertising, store placement, brand endorsements, and digital games can have considerable impact on consumer behavior. Norman et al. (2016), for example, reviewed effects of exposure to hedonic food information in children and found it to have a causal, dose–response effect on preferences, choices, and consumption of unhealthy foods. Vukmirovic (2015) reviewed effects of exposure to both hedonic and healthy information in adults and found that both types of information affected food preference, choice, and consumption.

Exposure to hedonic food information is likely to play a central role in the obesogenic food environment, amplifying the widespread consumption of unhealthy energy-dense foods that are palatable, socially acceptable, and inexpensive (Marteau et al. 2012). Although sources of health information encourage healthy eating, their influence may often fail to counteract the overwhelming effects of their unhealthy counterparts. In this context, overweight and obesity have become challenging public health issues worldwide, with high prevalence in both children and adults (Hales et al., 2017), accompanied by serious health consequences (GBD Obesity Collaborators, 2017). Additionally, most overweight and obese individuals cannot achieve and maintain significant weight loss (Knowler et al., 2009).

To better understand how exposure to food information affects eating behavior, it is important to establish the cognitive and affective processes that underlie food preference, choice, and consumption (cf. Sheeran et al., 2017). By establishing these processes, we can better understand the effects of exposure to hedonic and healthy food information in the environment, along with whom it affects most. We can also develop precision interventions that offset the effects of unhealthy food information and that enhance the effects of healthy food information. Here, we develop an experimental approach for examining these issues, motivated by memory research in cognitive psychology.

### Incidental acquisition of food information

Some food information may be learned intentionally and remembered deliberately, as when people learn and practice dieting. The acquisition and use of most food information, however, may often occur in a much more incidental and unintentional manner (e.g., Marteau et al., 2012; Papies, 2016a, b, 2017). When people encounter food information in the environment, it is unlikely that they intentionally try to establish memories of it. Although people may actively engage with this information as they process and evaluate it, they may not attempt to learn anything from it intentionally. Nevertheless, information from these processing episodes may become established in memory incidentally, especially when processed deeply (as well-designed food information is typically meant to be).

Classic memory research indeed demonstrates that extensive learning occurs incidentally as a byproduct of deep processing (e.g., Craik & Lockhart, 1972; Craik & Tulving, 1975; Hyde & Jenkins, 1973; Jacoby, 1983). As long as participants process a stimulus deeply for its meaning or self-relevance, they remember it well on later memory assessments, even though they had no idea that memory would be tested (e.g., Hamilton et al., 1980; Nairne et al., 2007; Roediger, 1990; Rogers et al., 1977). People often remember incidentally acquired information as well or better than information acquired intentionally. The implication is that a tremendous amount of information becomes established incidentally in memory. No doubt, much useful information is acquired in this manner, although detrimental information can be acquired as well (e.g., prejudice and stereotypes; Greenwald & Banaji, 1995, 2017). To the extent that food information is processed deeply, it is likely to leave long-term effects on memory, even though no intention existed to acquire it.

### Indirect activation and use of food information

Once food information has been acquired incidentally, it may become active unintentionally on later occasions when encountering related foods and deciding whether to purchase or consume them. Although no intention exists to retrieve and use this information, it becomes active involuntarily when encountering a relevant food and influences decision-making, especially when little explicit thought goes into the decision. Following a classic distinction in the memory literature, we assume that unintentionally activating previously acquired food information constitutes a form of *indirect memory*: Whereas direct memory occurs during a conscious deliberate attempt to remember something, indirect memory occurs when memories become active involuntarily in the absence of a conscious intention to remember (Johnson & Hasher, 1987; Richardson-Klavehn & Bjork, 1988).

Importantly, the distinction between direct and indirect memory tasks makes no assumptions about *underlying memory processes*. Potentially, both explicit memories and implicit memories can become active during each kind of task (where explicit memories are typically assumed to be conscious and effortful, and implicit memories are typically assumed to be unconscious and effortless; Johnson & Hasher, 1987; Richardson-Klavehn & Bjork, 1988). Although direct memory tasks primarily engage explicit memory processes, they may also engage implicit memory processes to a lesser extent. Although indirect memory tasks primarily engage implicit memory processes, they may also engage explicit memory processes occasionally. Thus, the indirect activation of food information when making food choices potentially includes both implicit and explicit memories. The paradigm developed here was not designed to establish which types of memory become active indirectly, nor does this issue bear on the claims we make. Instead, our primary claim is simply that foods activate memories indirectly, in turn affecting food preferences.

It is important to note that the indirect activation of incidentally acquired food information differs from classic priming effects that result from immediate contextual cues. Of interest in the experiment reported here is how *a food itself*—in the absence of contextual primes—indirectly activates incidentally learned information that affects its processing. We return to the distinction between incidental learning and health priming in eating research later.

Much memory research demonstrates that information in memory becomes active indirectly as people perform a broad spectrum of cognitive tasks (e.g., Corkin, 1968; Jacoby, 1983; Jacoby et al., 1989; Milner et al., 1968; Reber, 2013; Roediger, 1990; Roediger & McDermott, 1993; Schacter et al., 1993; Squire et al., 1993). Because this information is not required for task performance, it is not activated intentionally but instead becomes active involuntarily. Although some indirectly activated information may be experienced consciously, much of it often remains unconscious. Nevertheless, these indirect activations often have considerable impact, speeding the processing of perceptual stimuli, facilitating the execution of motor responses, and activating relevant semantic information.

Processing food information in the environment offers a paradigm case of the continual interaction between incidental learning and indirect memory. For example, after encountering food information that highlights hedonic features of cheeseburgers (e.g., tasty, savory, filling), later encountering a cheeseburger may indirectly activate memories of these incidentally established features, producing a hedonic simulation of enjoying the cheeseburger that motivates its consumption (Papies & Barsalou, 2015). Alternatively, after encountering food information that highlights a cheeseburger’s unhealthy features (e.g., high in fat, salt, and additives), later encountering a cheeseburger may indirectly activate memories of these features, producing simulations of unhealthy long-term consequences that inhibit consumption.

### Paradigm

In a novel well-controlled experimental paradigm, we assessed whether incidentally acquired memories of hedonic versus healthy food features affected food preferences indirectly a day later. We next provide an overview of this paradigm and our measure of food preference.

#### Assessing indirect effects of incidentally acquired food information

**Fig. 1:**
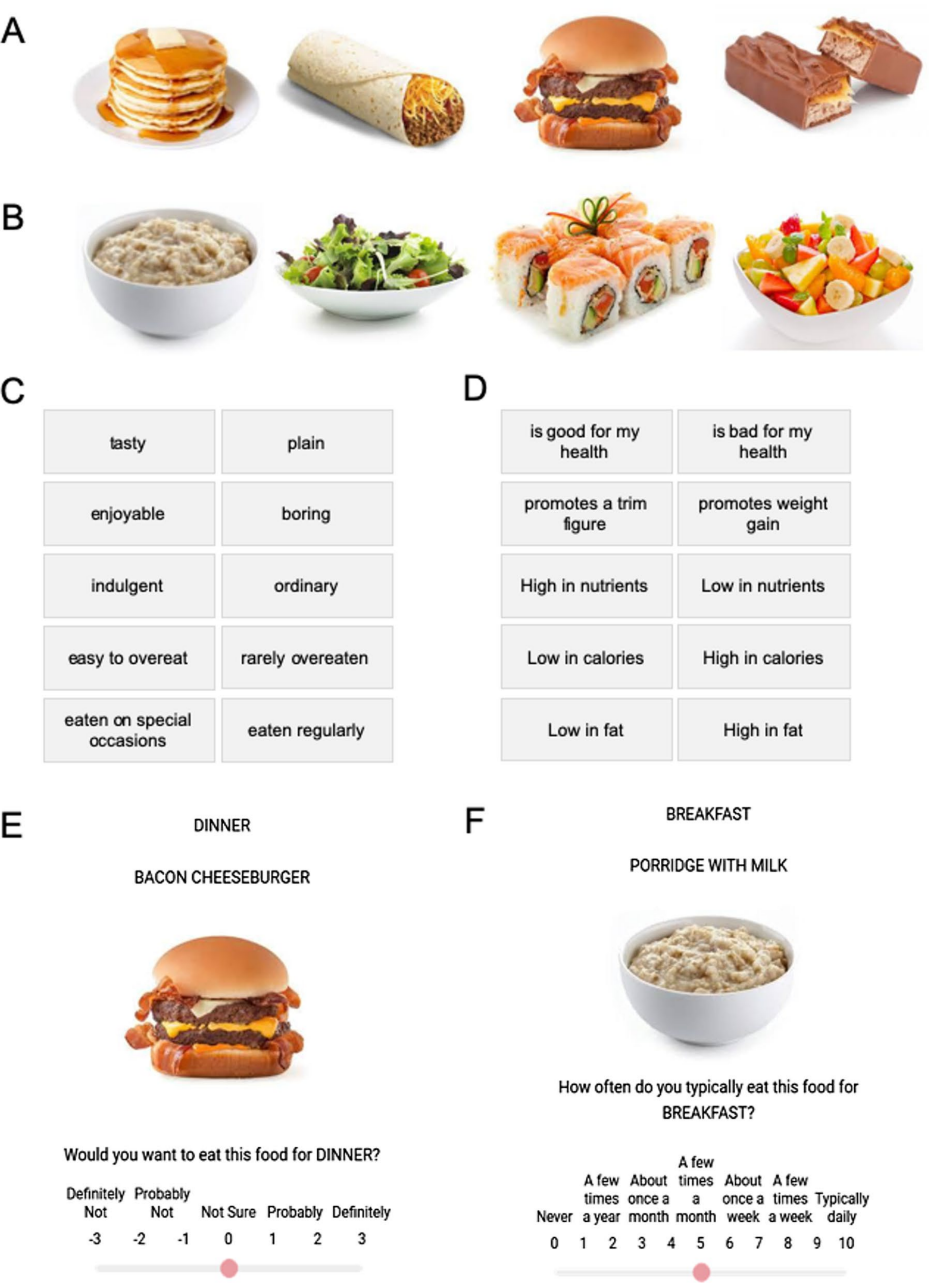
Examples of the tasty foods (**A**) and healthy foods (**B**) used in the experiment (see Additional file 1: Figures SM-1 and SM-2 for the complete food sets). Hedonic features (**C**) and healthy features (**D**) that participants could endorse for foods during the training phase. An example of a food preference trial (**E**). An example of a frequency trial for habitual food consumption (**F**)

During an initial incidental learning procedure, one group of randomly assigned participants was exposed to hedonic features of 24 tasty foods and 24 healthy foods (the hedonic exposure group). A second group of participants was exposed to healthy features of the same 48 foods (the health exposure group). Figure 1A and B illustrate examples of the tasty and healthy foods. Figure 1C presents the hedonic features that the hedonic exposure group received, and Fig. 1D presents the healthy features that the health exposure group received. In what was presented as a consumer feedback task, the hedonic exposure group endorsed the hedonic features that they perceived in each food, and the health exposure group endorsed the healthy features that they perceived in each food. Participants in both groups were led to believe that they were simply evaluating the features of foods in a consumer survey, with nothing said about learning or a later memory assessment. Thus, the endorsement task created an incidental learning manipulation between groups, with the hedonic group exposed to hedonic features, and the health group exposed to healthy features.

Similar to how food information in the environment is often presented to consumers, the endorsement task made food features salient and actively engaged participants in processing them deeply. As people encounter food information, they may evaluate it, discuss it with others, and make decisions about purchasing or consuming specific foods. Establishing the features that a food does or does not have is an important part of this process, captured by the endorsement task in our exposure manipulation.

One day later—after processing associated with incidental learning had subsided—participants performed a food preference task (Fig. 1E). On each trial, participants were asked how much they would want to eat each food for a particular meal (e.g., How much would you want to eat FISH AND CHIPS for DINNER?). Of interest was whether information acquired incidentally the day before during exposure became active indirectly to affect food preferences.

To assess whether either hedonic exposure, health exposure, or both types of exposure affected food preference relative to a baseline, a third group of participants performed the preference task with no previous exposure (the no-exposure baseline group). The three different types of exposure were implemented between groups to minimize demand and repetition effects that would have complicated interpretations of a repeated measures design. A no-exposure baseline was used because it offers the most naturalistic approach to assessing whether exposure to hedonic and healthy food information affects food preferences.^1^ Of interest in the real world is how exposure to new food information changes food preferences relative to the steady state of current food knowledge (for discussion of other possible baselines, please see footnote 1).

To further minimize demand while participants made food preference judgments, a comprehensive cover story obscured the relation between the incidental learning and food preference tasks. As a consequence, participants had no reason to intentionally learn or deliberately remember information from the exposure phase. Instead, if information from the exposure phase affected food preferences later, it is likely to have done so indirectly.

We assessed exposure effects relative to people’s eating habits. One possibility is that exposure to hedonic and healthy features affects preferences for all foods, regardless of whether they are consumed frequently or infrequently. Another possibility is that exposure has relatively little impact on frequently consumed foods. Because eating habits have much more strength in memory than information acquired incidentally via brief exposure, eating habits could dominate preference. If so, then exposure effects should primarily occur for foods consumed infrequently, given the greater potential for influencing their preferences. Much related work demonstrates the powerful ability of habits to override other sources of influence in cognition and behavior (Mazar & Wood, 2018; Orbell & Verplanken, 2018; Ouellette & Wood, 1998; Webb & Sheeran, 2006). To assess these possibilities, our paradigm collected data on how frequently participants consumed the 48 foods (in an additional consumer survey that followed the food preference task; Fig. 1F).

Finally, we assessed whether exposure effects interact with individual differences. Much research reports that individual differences interact with interventions to change eating habits (e.g., Buckland et al., 2018). We therefore included measures of healthy eating habits, dietary restraint, and body mass index (BMI)^2^ to assess whether these individual differences moderated any observed exposure effects. It is important to note that even though BMI is not a perfect indicator of health, it nevertheless remains strongly associated with unhealthy eating behavior, body fat, and poor health outcomes in eating research (please see footnote 2 for further details).

#### Assessing food preference

As just described, our experiment assessed the impact of exposure to hedonic versus healthy features on food preferences for tasty versus healthy foods. We could have measured these preferences in the preference phase by simply asking participants to indicate their overall preference for tasty foods and their overall preference for healthy foods (as often done in the literature; e.g., Hearty et al., 2007). Much work shows, however, that general decontextualized assessments often fail to predict behavior well. Instead, *focused assessments in specific situations* are more accurate (e.g., Ajzen & Fishbein, 1977, 2005; Glasman & Albarracín, 2006; Siegel et al., 2014).

For this reason, we focused the assessment of food preference in two ways. First, instead of assessing each participant’s overall preference for tasty and healthy foods, we assessed their preference for each of 24 specific foods within each food type. Additionally, instead of assessing a participant’s general preference for a specific food, we asked them how much they would want to eat the food for a specific meal (e.g., how much would you want to eat PIZZA for DINNER?). By specifying both specific foods and specific meals, we focused participant’s judgments on specific eating situations.

Measures of food preference typically correlate with consumption (e.g., Boswell et al., 2018; Hollands & Marteau, 2016; Van Dessel et al., 2018; also see Norman et al., 2016; Vukmirovic, 2015). As authoritative reviews by Subar et al. (2015) and Thompson and Subar (2017) describe, self-report measures often provide accurate estimates of consumption, especially when the goal is not to estimate energy intake precisely in terms of calories, but is instead to assess the foods consumed. Indeed, these reviews document the importance of self-report eating instruments in health and nutrition science. To establish the validity of our food preference measure, we demonstrate later that it tracks predicted differences in BMI and healthy eating habits. We also discuss the importance of assessing food preferences and eating intentions during the preliminary phases of eating prior to consumption (also see Sobal et al., 2014).

### Experiments performed

Using the paradigm just described, we performed three experiments that assessed the indirect effects of incidental exposure to food information. The first was a small pilot experiment that offered a preliminary assessment of hedonic and health exposure effects and their interaction with eating habits without the no-exposure baseline (*n* = 39). As predicted, the pilot experiment found that: (1) manipulating hedonic versus health exposure affected food preference, and (2) exposure interacted with eating habits. We do not report the pilot experiment’s results here but provide a document on OSF that provides a complete account of its methods and results (https://osf.io/y2zpk/).

Based on the pilot experiment, we subsequently ran two identical pre-registered experiments with larger samples that attempted to replicate the pilot experiment’s critical findings (*n* = 302, *n* = 315). Besides replicating the basic design of the pilot experiment, these second and third experiments added the no-exposure baseline and assessed individual difference measures for BMI, healthy eating habits, and eating restraint.

The pre-registration for the second experiment formalized the informal predictions in the pilot experiment and added new predictions for the exposure baseline and individual difference measures (https://osf.io/re5mw/). The second experiment’s results replicated the pilot experiment’s key findings and partially confirmed the new predictions for the no-exposure baseline and individual difference measures. Because these new predictions were only partially confirmed, we wanted to replicate the pattern of results obtained.

We therefore used the second experiment’s results to make pre-registered predictions for the third experiment (https://osf.io/aes79/). Because the second and third experiments were identical, our intention at that time was to eventually combine them. Thus, the second preregistration predicted, first, that the third experiment would replicate the second experiment’s results, and second, that when we combined these two experiments, the combined results would demonstrate the second experiment’s results with greater power (https://osf.io/aes79/).

As predicted, the second and third experiments produced the same general pattern of predicted results. To simplify presentation, we only present results from the combined experiment here and only address its pre-registered predictions. For interested readers who would like to compare results across the two parts of the combined experiment, the individual results can be found in Additional file 1, the Supplemental Material (SM), referred to there as Parts A and B.

To further streamline presentation, only the primary hypotheses in the combined experiment’s pre-registration are addressed here.^3^ All hypotheses not addressed were secondary (a complete list of these hypotheses and their results can be found in footnote 3). A document that presents all pre-registered hypotheses for Part A, Part B, and the combined experiment can be found on the project’s OSF site, together with the specific results that bear on each (https://osf.io/y2zpk/).

### Hypotheses

The hypotheses presented next were the central ones preregistered on OSF for the combined experiment. For readers interested in the underlying theoretical motivation for these hypotheses, detailed explanations for specific predictions can be found in an additional document on OSF (https://osf.io/y2zpk/; for a general account, see Papies & Barsalou, 2015).

**Hypothesis 1: Effects of hedonic versus health exposure on food preference**

Incidental exposure to hedonic versus healthy food features will indirectly influence preferences for tasty versus healthy foods a day later. Although tasty foods will be preferred more than healthy foods in *both* the hedonic and health exposure groups (a main effect), tasty foods will become even more preferred relative to healthy foods following hedonic exposure than following health exposure (an exposure × food type interaction on food preference).

Should this predicted interaction occur, it follows that incidental learning affects food preferences indirectly a day later. If incidental learning has no effect, the two exposure conditions should not differ. Furthermore, because the two exposure conditions are well-matched for content and tasks during training (day 1), any observed difference between them rules out the possibility that effects on food preference (day 2) only reflect simple fluency (Jacoby, 1983; Jacoby et al., 1989). If only fluency were operating, the two exposure conditions would not show different patterns for food preference. Instead, differential exposure effects most likely result from differential processing of food features during the endorsement task (please see footnote 1 for further discussion).

**Hypothesis 2: Effects of hedonic versus health exposure relative to the no-exposure baseline**

Relative to the no-exposure baseline, only hedonic exposure will produce a group-level effect on food preferences. Although tasty foods will be preferred more than healthy foods for *both* the hedonic and no-exposure groups (a main effect), tasty foods will be even more preferred relative to healthy foods following hedonic exposure than following no exposure (an exposure × food type interaction on food preference). Because hedonic exposure activates common affective processes associated with pleasure and reward across individuals, it will generally influence individuals regardless of their BMI and healthy eating habits.

In contrast, health exposure will not exhibit a group-level effect of on food preferences. Because health exposure is most likely to only influence food preferences for individuals concerned about their body weight (e.g., individuals with high BMI), health exposure will not produce a group-level effect (for related results, see Buckland et al., 2018; Papies, 2016a, 2016b). As predicted in Hypothesis 5, however, health exposure will influence food choices for high-BMI individuals (i.e., an individual-level effect).

**Hypothesis 3: Effects of hedonic and healthy endorsements on food preference**

As just predicted, we only expected hedonic exposure to have an overall effect on food preferences—not health exposure. An explanation of this potential effect is that only making hedonic endorsements during the exposure phase affected food preferences later—making healthy endorsements did not.

Specifically, we reasoned that as more hedonic features become active and endorsed during hedonic exposure, more pleasure will be anticipated from consuming the food being evaluated (Papies & Barsalou, 2015). In the process of simulating these pleasure experiences, robust memories will be established incidentally. When participants perform the food preference task the next day, these robust memories will be highly available and become active indirectly to affect task performance. As the number of hedonic features in an activated memory for a food increases, preference for consuming the food will become stronger, reflecting greater anticipated pleasure.

In contrast, we expected that health exposure would produce relatively “cold” cognitive appraisals of foods that lack the “hot” affective elements associated with hedonic pleasure and reward (Metcalfe & Mischel, 1999; Strack & Deutsch, 2004). As a consequence, memories established incidentally during health exposure will not be as robust and accessible as memories established during hedonic exposure. As a further consequence, these memories will be less likely to become active and affect food preferences.

To establish whether only hedonic endorsements affected food preferences, we predicted the presence of an exposure × endorsements interaction (Hypothesis 3). Specifically, we predicted that preferences for foods would only increase as more hedonic features were endorsed for them, not as more healthy features were endorsed.

**Hypothesis 4: Effects of consumption frequency on food preference**

As foods are consumed more frequently, preferences for them will increase substantially across all exposure conditions (a main effect of consumption frequency). In other words, eating habits will produce a large frequency effect on current food preferences.

Additionally, because eating habits have much more strength in memory than information acquired incidentally during brief exposure, eating habits will dominate preference relative to endorsement. As a consequence, the endorsement effect predicted for Hypothesis 3 will become minimal at high levels of consumption frequency (a frequency × endorsement interaction on food preference)—increasing consumption frequency will attenuate the effect of increasing endorsement.

Finally, because we only predict an endorsement effect for hedonic exposure (Hypothesis 3), the attenuating effect of consumption frequency on endorsement will primarily occur for hedonic exposure (an exposure × frequency × endorsement interaction on food preference).

**Hypothesis 5: BMI modulates the effect of health exposure**

As described for Hypothesis 2, we did not expect health exposure would have a general effect on food preferences across individuals (relative to the no-exposure baseline). Instead, we hypothesized that health exposure would only influence food preferences for individuals who are likely to be concerned about their body weight (e.g., individuals high in BMI; for related results, see Buckland et al., 2018; Papies, 2016a, b). Rather than predicting a group-level effect of health exposure, we predicted an individual-level effect. Specifically, health exposure will only affect individuals high in BMI (relative to the no-exposure baseline), decreasing their overall preference for all foods, regardless of whether foods are tasty or healthy (a health exposure × BMI interaction on food preference).

We reasoned that during health exposure, healthy features of food will become salient and important for many high-BMI individuals. As a consequence, robust memories of food healthiness will become established in memory incidentally for them. When these individuals perform the food preference task the next day, these robust memories will be highly available and become active to affect preferences indirectly. Specifically, these individuals will adopt a restrained perspective on food consumption that reduces their overall preference for both food types. Consistent with reducing overall calorie intake, these individuals will temper their interest in all foods, both tasty and healthy.

## Methods

The Ethics Committee of the College of Science and Engineering at the University of Glasgow approved this research. All experimental materials are available in the SM and on OSF (https://osf.io/ys4q2/).

### Design

To avoid demand and repetition effects, exposure was manipulated between three exposure groups (not as repeated measures): hedonic exposure, health exposure, and no exposure. During the exposure phase on day 1, participants in the hedonic and health exposure groups endorsed each of the same 48 foods (24 tasty, 24 healthy) for either its hedonic features (hedonic exposure) or for its healthy features (health exposure). One day later, participants in all three exposure groups performed a test session on the 48 foods from day 1 (identical across groups). The test session included: (1) a food preference task; (2) a consumption frequency task; (3) collection of individual difference measures (BMI, healthy eating habits, dietary restraint); (4) assessment of experimental demand.

Overall, the experimental design included one independent variable manipulated at the group level (exposure), another manipulated within participants (food type), and five continuous predictors (endorsements, consumption frequency, BMI, eating habits, restraint). Food preference served as the primary dependent variable. Foods and participants were included as random effects.

### Participants and sample size

Following the pilot experiment, we performed a power analysis to establish suitable power for Part A of the combined experiment (described in the SM). Based on this analysis, Part A included 302 participants assigned randomly to hedonic exposure (*n* = 102), health exposure (*n* = 100), and no-exposure (*n* = 100). Part B provided a replication of Part A that included 315 participants assigned randomly to hedonic exposure (*n* = 103), health exposure (*n* = 105), and no-exposure (*n* = 107). When Parts A and B were combined, the hedonic, health, and no-exposure groups contained 205, 205, and 207 participants, respectively, (617 total). All participants in Parts A and B were recruited from the Prolific online platform, which had a panel of over 50,000 individuals at the time (paid £6/h). Requirements for participation included age (18–30), minimum number of previous Prolific studies (10), minimum Prolific approval rate (95%), language fluency (English), and residence (a current UK resident). Because the pilot experiment developed a sample of foods relevant for UK participants aged 18–30, both later experiments sampled the same age group as well.

No participant who completed both sessions in any of the three experiments was excluded. In the pilot experiment, all participants completed both sessions. In Parts A and B, 3 and 8 participants, respectively, did not complete both sessions and were not included in the sample sizes just reported.

### Materials

For each of 4 eating situations (breakfast, lunch, dinner, snack), 6 tasty and 6 healthy foods were sampled from previously established food norms (Werner et al., 2021). In these norms, the 24 tasty foods were highly rated for tastiness and fillingness but not for healthiness, whereas the 24 healthy foods exhibited the opposite pattern. Later results verify our pre-registered prediction that hedonic and healthy endorsements would confirm the assignments of foods to the tasty and health food groups.

To support the experimental cover story, 6 hedonic and 6 healthy birthday gifts were also included (e.g., cocktail making master class, fitness tracker wristband). The SM provides a complete set of the food and gift stimuli presented across experiments.

### Day 1 procedure

After being recruited on the Prolific platform, participants were directed to the Qualtrics platform, where they performed the first session online. Participants were informed that their data would be completely anonymous and that we would have no access to their personal data. Participants were then told that they would perform a series of consumer surveys across two sessions on two consecutive days and provided consent. At multiple points, the instructions conveyed that there were no correct answers to any of the questions that participants would be asked and that instead we wanted to know how *they* perceive the qualities and desirability of consumer products. We further asked them to answer intuitively with whatever came to mind naturally without a lot of thought. Once participants read the instructions, they were asked to work in a quiet place where there were no distractions. They were also asked to not take any breaks.

To prevent demand, participants were led to believe that the individual surveys in the two sessions were unrelated. Specifically, participants were told that we were surveying products from multiple consumer categories, including cars, clothing, electronics, foods, and gifts, and that, across a series of surveys, products from these categories would be assessed for a variety of their qualities and desirability. Participants were then told that they had been selected to evaluate products from the categories of gifts and foods. To introduce participants to the first survey, they received an example of a gift (juicer) with 10 features arranged in 2 columns below. Participants in the hedonic exposure group received 5 pairs of hedonic/non-hedonic properties; participants in the health exposure group received 5 pairs of healthy/unhealthy properties. The 10 endorsement features in each exposure condition were constant across gifts. Nothing was said to participants about whether the features they assessed were hedonic or healthy, and they knew nothing of the other exposure group. They simply received the 10 features below each gift and were asked to tick off as many or as few of the features that they believed applied to it. Specifically, they were asked, “From the list below, please select all the qualities that you think apply to this gift.”

Participants then received an example of a food (Victoria sponge cake), with analogous instructions to tick off as many or as few of the features that they believed applied to the product. Figure 1C presents the 10 features that the hedonic exposure group assessed; Fig. 1D presents the 10 features that the health exposure group assessed (constant across foods). Again, nothing was said to participants about whether the features they assessed were hedonic or healthy. Participants again had no idea that another group of participants was assessing different features for the foods.

Similar to the presentation of much food information in the environment, the endorsement task made food features salient and actively engaged participants in processing them. This task also had several useful properties for implementing incidental learning: (1) It ensured deep processing of the foods, similar to orienting tasks often used to implement depth-of-processing manipulations in the memory literature. (2) It made hedonic or healthy food features salient. (3) It provided a cover story that blocked intentional learning. (4) It allowed us to establish that the 24 tasty foods were indeed high in hedonic features and low in healthy features, and conversely, that the 24 healthy foods were high in healthy features and low in hedonic features.

Participants then performed these two surveys. During the first, they received 12 gifts from the category of birthday gifts (with the category mentioned explicitly). Although these trials served as practice, participants were not aware of this, but instead believed that we were collecting consumer evaluations of gifts. The 12 gifts were randomized differently for each participant.

Participants then received the 48 foods, which were similarly presented in blocks of 12 for breakfast foods, lunch foods, dinner foods, and snack foods. These blocks were presented in a fixed order to reflect the temporal order in which meals normally occur over the course of a day (snacks were presented last, given that they could occur any time). The relevant eating situation was labeled explicitly prior to each block, with its 12 foods randomized for each participant.

Once participants finished evaluating the 48 foods, they were immediately asked to repeat the two surveys just performed (i.e., thereby doubling the amount of exposure received). To justify these repeated tasks to participants, we told them that performing the surveys again would increase the accuracy of our product assessments. Specifically, participants were told that, “Previous research has found that people's evaluation of consumer products improves with practice. For this reason, we would like you to evaluate the products again one more time. This will help us establish the perceived qualities of these products as best as possible.” Participants then evaluated the same 12 gifts and 48 foods as before, with the products in each block shown in a new random order.

Once participants completed these last two blocks, they were told that further consumer surveys would follow the next day. Participants had no reason to believe that they needed to learn information during the exposure phase, such that any information acquired was learned incidentally (not intentionally). The day 1 session took approximately 20 min. All participants performed this session on the same day, within a few hours of recruitment messages being distributed.

#### Endorsement scores

We computed an endorsement score for a participant’s assessment of each food. For hedonic exposure, the number of non-hedonic features endorsed (Fig. 1C, right) was subtracted from the number of hedonic features endorsed (Fig. 1C, left) to create an endorsement score for each food from -5 to + 5 (non-hedonic to highly hedonic). For health exposure, the number of unhealthy features endorsed (Fig. 1D, right) was analogously subtracted from the number of healthy features endorsed (Fig. 1D, left), to create an endorsement score for each food from − 5 to + 5 (highly unhealthy to highly healthy). A participant’s overall endorsement of a food was calculated as the average of the endorsement scores from its two presentations.

### Day 2 procedure

Twenty-four hours after the day 1 session, all participants in the hedonic exposure and health exposure groups received a second anonymous link via Prolific that redirected them to the Qualtrics platform for the day 2 session. Concurrently, additional participants were recruited from Prolific for the no-exposure group, who met the same inclusion criteria as the participants recruited into the exposure groups. Participants in all groups were required to perform the day 2 session by midnight.

Participants were informed that they would perform a series of consumer surveys. Again, they were told that we were interested in how people perceive products in various consumer categories (cars, clothes, electronics, foods, gifts), and that they had been chosen to evaluate gifts and foods. Nothing indicated to the exposure groups that session 2 was related to session 1 in any way. Instead, the day 2 session was simply described as further consumer surveys. To the extent that any information learned incidentally became active during session 2, it became active indirectly. Rather than being asked to deliberately remember information, participants were simply asked to provide food preferences and frequency estimates. Thus, any information from session 1 that became active was indirect in the sense of not being requested explicitly or necessary for performing the current tasks.

Participants first performed a preference task on the 12 birthday gifts from the day 1 exposure session, randomly ordered for each participant. For each gift, they were asked, “Would you want to give this as a BIRTHDAY GIFT?” Participants responded on a -3 to + 3 continuous slider with the labels: *definitely not, probably not, not sure, probably, definitely* (positioned initially at 0). Participants then performed a preference task on the 48 foods from the day 1 exposure session in explicitly labeled blocks for breakfast, lunch, dinner, and snack (in this fixed order, with the 12 foods in each block randomly ordered for each participant). For both preference tasks, participants were told that we were interested in the desirability of products from the two consumer categories. Prior to the gift block, participants practiced on 1 gift and 1 food.

For the food preference task, participants were asked, “Would you want to eat this food for [MEAL]?”, where [MEAL] could be BREAKFAST, LUNCH, DINNER, or SNACK (Fig. 1E). We selected this wording because it is sufficiently ambiguous to motivate preferences based on either hedonic or healthy features. Participants could want to eat a food because it would be pleasurable or because it would be healthy. Indeed, phrasing the task as “Would you want to eat this food…” implies wanting something because of its incentive value, which can take many different forms, including hedonic pleasure and healthy outcomes (Berridge, 1996; Berridge et al., 2009).

To make each food preference judgment, participants clicked the point on the slider scale that best represented how much they would want to eat the food for a particular eating situation. The more likely they were to eat a food, the more they should click a point on the scale toward + 3. The less likely they were to eat the food, the more they should click a point toward − 3. The more unsure they were about whether or not to eat the food, the more they should click a point near 0. The preference slider was always positioned at 0 initially.

After completing the gift and food preference trials, participants were told that a final consumer survey would ask them to estimate how often they give each gift as a birthday present, and how often they eat each food for a meal. Participants received the 12 gifts in a random order and rated each on a 0 to 10 continuous slider scale for “How often do you give this as a BIRTHDAY GIFT?” with scale labels ranging from “Never” to “Every time.” They then received the 48 foods and rated each on a 0 to 10 continuous slider scale for “How often do you typically eat this food for [MEAL]?” with scale labels from “Never” to “Typically daily.” Figure 1F presents the screen format and slider scale for the assessment of food consumption frequency, with the slider always positioned initially at 5. Again, foods were blocked by meals in a fixed order (breakfast, lunch, dinner, snack), with the 12 foods randomized within each block for each participant.

Following the frequency assessments, demand was assessed with a series of four quantitative items: (1) To what extent did your responses to the survey questions reflect your personal assessments of the products you viewed? (2) To what extent did you try and respond in a way that you thought the survey researchers wanted to hear? (3) To what extent did you respond intuitively and naturally to the survey questions without a lot of deliberate thought? (4) To what extent do you believe that there are correct answers to the survey questions? Participants responded to all four questions using a 0 to 6 continuous slider scale with the labels: Not at all, Moderately, Completely.

Individual difference measures were then collected. To establish BMI, we asked participants for their height and weight (without mentioning that BMI was being assessed). To assess healthy eating habits, we asked participants to complete the Adolescent Food Habit Checklist (Johnson et al., 2002). To assess eating restraint, we asked participants to complete the restraint scale of the Three Factor Eating Questionnaire (TFEQ-R18; Anglé et al., 2009).

Finally, participants were debriefed, thanked, and redirected back to the Prolific platform for payment. Qualtrics screens for the survey can be found on OSF (https://osf.io/ys4q2/).

### Regression analysis procedure

The primary goals of our analysis procedure were to: (1) identify likely effects, (2) establish their effect sizes, and (3) assess their generalizability across participants and foods. To do so, we first *z*-transformed the dependent variable and its predictors to specify each predictor’s effect in standard deviation units. As a consequence, each estimated regression coefficient indicates the standard-deviation-unit change in the dependent variable associated with each standard-deviation-unit change in the respective predictor. The sign of these standardized coefficients indicates the direction of the relationship. If, for example, a standardized coefficient for the relation between consumption frequency and food preference happened to be 0.50, this meant that food preference increased positively by 0.50 of a standard deviation for each standard deviation increase in consumption frequency. The larger a coefficient, the larger its effect size.

In each regression analysis, we implemented a sequence of three multilevel mixed-effect models (using the lme4 package in R; Bates et al., 2015). We will refer these models as *Model 1*, *Model 2*, and *Model 3*. These models were *multilevel* because they predicted a dependent variable such as food preference using both food-level predictors (endorsements, consumption frequency) and individual-level predictors (BMI, healthy eating habits). These models were *mixed effect* because they simultaneously assessed both fixed effects (exposure, food type) and random effects (random intercepts for participants and foods; random slopes that captured variability in the fixed effects across participants and foods). Assessing random effects is essential for generalizing results beyond participants and foods in the current samples (Barr et al., 2013). Mixed-effect modeling offers a powerful approach for establishing the generalizability of effects across participants and foods simultaneously.

In the first stage of our analysis procedure, Model 1 identified predictors (main effects and interactions) likely to have meaningful effects on the dependent variable. Model 1 included main effects for all predictors of interest at the participant and food levels, all interactions of these predictors up through three-way, and random intercepts for participants and foods. This relatively liberal model served to identify potentially important predictors that were subsequently examined more closely and conservatively in Models 2 and 3. For a main effect or interaction to pass this initial screening, the *t* for its estimated regression coefficient had to be greater than |1.96| (associated with a *p* value ≤ 0.05). We assumed that any effect that failed this relatively liberal initial screening would be unlikely to have a meaningful impact on the dependent variable.

For each potentially important effect identified in Model 1, we then assessed it more conservatively in a unique Model 2 that tested it *maximally* (Barr et al., 2013). Specifically, maximal testing established whether an effect in Model 1 generalized across participant-level and food-level variability in the current sample, and also whether it is likely to generalize across future samples of participants and foods. Imagine, for example, that a 0.50 estimated regression coefficient for consumption frequency survived initial screening in Model 1. If large individual differences in participants and habits were associated with this effect, then it might not generalize to the broader populations of participants and foods. To test an observed effect in Model 1 maximally, Model 2 added one empirically determined random slope for each participant that modeled the effect for that participant. Additionally, Model 2 added one empirically determined random slope for each food that modeled the effect for that food. Of interest was whether the *t* for the effect in Model 2 remained greater than |1.96| once the variances of all random effects for participants and foods were accounted for simultaneously (i.e., both intercepts and slopes). If the effect passed this maximal testing, we concluded that it generalizes both in and beyond the participants and foods sampled here. If the effect failed maximal testing, we assumed that it does not.

Including appropriate random slopes simultaneously in Model 2 for each and every predictor that survives initial screening in Model 1 is typically not possible, as the sheer complexity of the model disrupts optimization and convergence. To circumvent this problem, Barr et al. (2013, p. 276) suggested maximally testing each effect of interest one at a time (i.e., including appropriate random slopes for foods and participants associated with the effect of interest, while not including random slopes for any remaining effects). Thus, when maximally testing the effect of (say) consumption frequency, a unique Model 2 was constructed by adding random slopes for consumption frequency to Model 1 but not adding random slopes for any other effect. In this manner, a unique Model 2 was constructed for each effect that passed Model 1 screening. Importantly, whenever a higher-order *interaction* passed Model 1 screening, random slopes were also included for all lower-order interactions and main effect terms nested within it (see Barr et al., 2013).

If an effect passed maximal testing in Model 2, it was evaluated one more time in a unique Model 3 that established how much unique variance it explained in Model 2. In each Model 3, we dropped the main effect or interaction being tested from its Model 2, along with any interactions containing it and any associated random slopes, while keeping everything else the same as in Model 2. We then subtracted the total variance for the effect’s Model 3 from the total variance for its Model 2. The difference in *R*^2^ (Δ*R*^2^ expressed as a percentage) established how much unique variance the effect captured when included *as a fixed effect together with associated interactions and random effects* in Model 2.

Using this analysis procedure, we established effects that generalized across the current samples of participants and foods, and that are also likely to generalize across future samples. For each effect established as generalizable in Model 2, we obtained two measures of its effect size: (1) its standardized regression coefficient in Model 2, and (2) its Δ*R*^2^ derived from Model 3.

## Results

Only results from the combined experiment are reported here. As described earlier, individual results for Parts A and B can be found in the SM, and results for the pilot experiment can be found on OSF (https://osf.io/y2zpk). The data and R analysis scripts for the combined experiment can also be found on OSF, along with those for Part A, Part B, and the pilot (https://osf.io/s5u3p). For interested readers, the SM also provides the average non-standardized measures for endorsement, preference, and consumption frequency in the combined experiment, for each of the 48 foods, in each exposure condition.

### Preliminary analyses

We first present the results of two preliminary analyses. The first provided a manipulation check of food type, demonstrating that the tasty and healthy foods varied in hedonic and healthy features as predicted. The second assessed the validity of our food preference measure, demonstrating that it reflected predicted differences in BMI and healthy eating habits. Each of these two analyses assessed (and verified) pre-registered predictions for the combined experiment.

#### Validation of the tasty versus healthy foods manipulation

The endorsement scores collected during the day 1 session offered a manipulation check of the tasty versus healthy food assignments. We predicted that the tasty foods would be endorsed as having many hedonic features and few healthy features. Conversely, we predicted that the healthy foods would be endorsed as having many healthy features and few hedonic features.

As described earlier, a single endorsement score resulted from how many hedonic or healthy features a participant endorsed for a food on each trial of the exposure phase, with the scores for the two presentations of the same food averaged across exposure blocks. In the hedonic exposure condition, increasing endorsement scores indicated that a food was perceived as increasingly hedonic. In the health exposure condition, increasing endorsement scores indicated that a food was perceived as increasingly healthy.

**Fig. 2:**
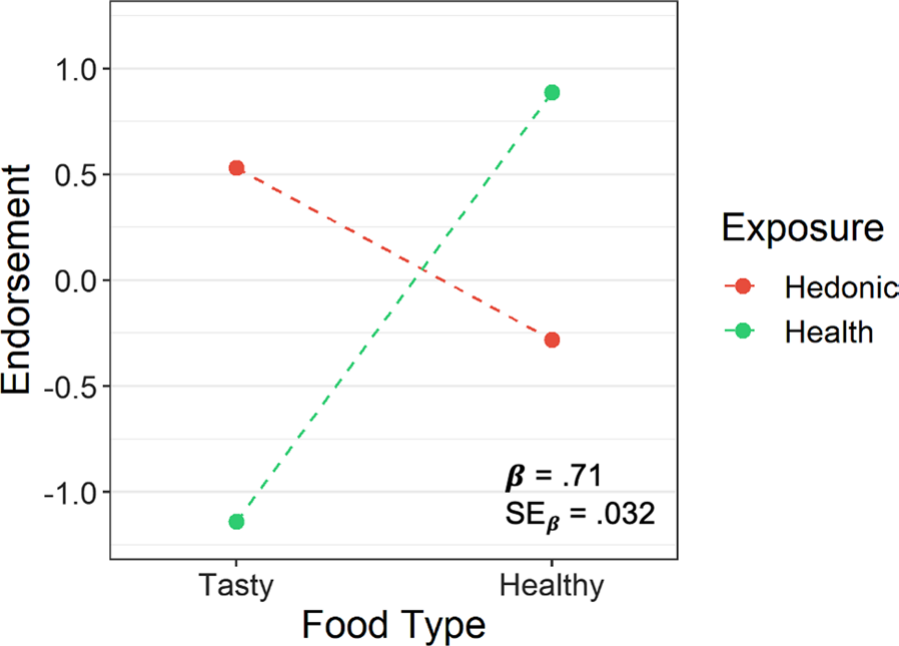
Evidence for the validity of the food type manipulation between tasty and healthy foods. Whereas tasty foods were high on hedonic endorsements and low on healthy endorsements, healthy foods were low on hedonic endorsements and high on healthy endorsements. Hedonic endorsements were produced in the hedonic exposure condition, and healthy endorsements were produced in the health exposure condition. A modeled interaction from regression is shown, with the endorsement scale plotted in standardized units. From Table 1, the standardized estimated regression coefficient for the interaction is shown (*β*), together with its standard error to provide a measure of expected variability (SE_β_)

To assess the validity of our tasty and healthy food assignments, endorsement scores were regressed onto exposure condition and food type. Figure 2 plots the results, and Table 1 presents the statistical analysis. As predicted, tasty foods received high hedonic endorsement scores from participants in the hedonic exposure condition, while receiving low healthy endorsement scores from participants in the health exposure condition. Conversely, healthy foods received low hedonic endorsement scores from participants in the hedonic exposure condition, while receiving high healthy endorsement scores from participants in the health exposure condition. As the interaction in Fig. 2 further illustrates, tasty and healthy foods differed much more in how healthy they were perceived than in how hedonic they were perceived (i.e., the slope for healthy endorsements was much steeper than the slope for hedonic endorsements). Participants clearly distinguished the relative healthiness of the tasty versus healthy foods.

**Table 1:** Mixed-effect regressions of endorsement on food type and exposure

DV: endorsement	Model 1			Model 2					Model 3	
Predictor	Estimate	SE	*t*	Estimate	SE	*t*	*R*^2^	AIC	∆*R*^2^	AIC
Food type	− .30	.028	− 10.78	− .30	.030	− 10.05	73	32,045	− 5	34,454
Exposure	.13	.010	12.70	.13	.031	4.01	73	31,779	− 4	34,531
Food type × exposure	.71	.004	176.94	.71	.032	22.29	78	28,847	− 61	53,007

Regressions were performed on standardized measures. Thus, an estimate is the estimate of a standardized regression coefficient in the respective model, with SE and *t* being the standard error and *t* value of the estimate. *R*^2^ is the total variance explained by Model 2, and ∆*R*^2^ is the amount of variance explained by the main effect or interaction dropped in Model 3 (both in percentages. AIC is the value of the Akaike Information Criterion for Models 2 and 3. For Food Type, tasty foods were coded + 1, and healthy foods were coded − 1. For Exposure, hedonic exposure was coded + 1, and health exposure was coded − 1

As Table 1 illustrates, the exposure × food type interaction in Fig. 2 passed maximal testing in Model 2, exhibiting a robust estimated regression coefficient (*t* >|1.96|). As a consequence, this predicted interaction generalizes across foods and participants here, and is likely to generalize across future foods and participants. Additionally, this interaction explained 61% unique variance associated with endorsement scores in Model 3, indicating that tasty and healthy foods differed substantially as expected.

#### Validation of the food preference measure

**Fig. 3:**
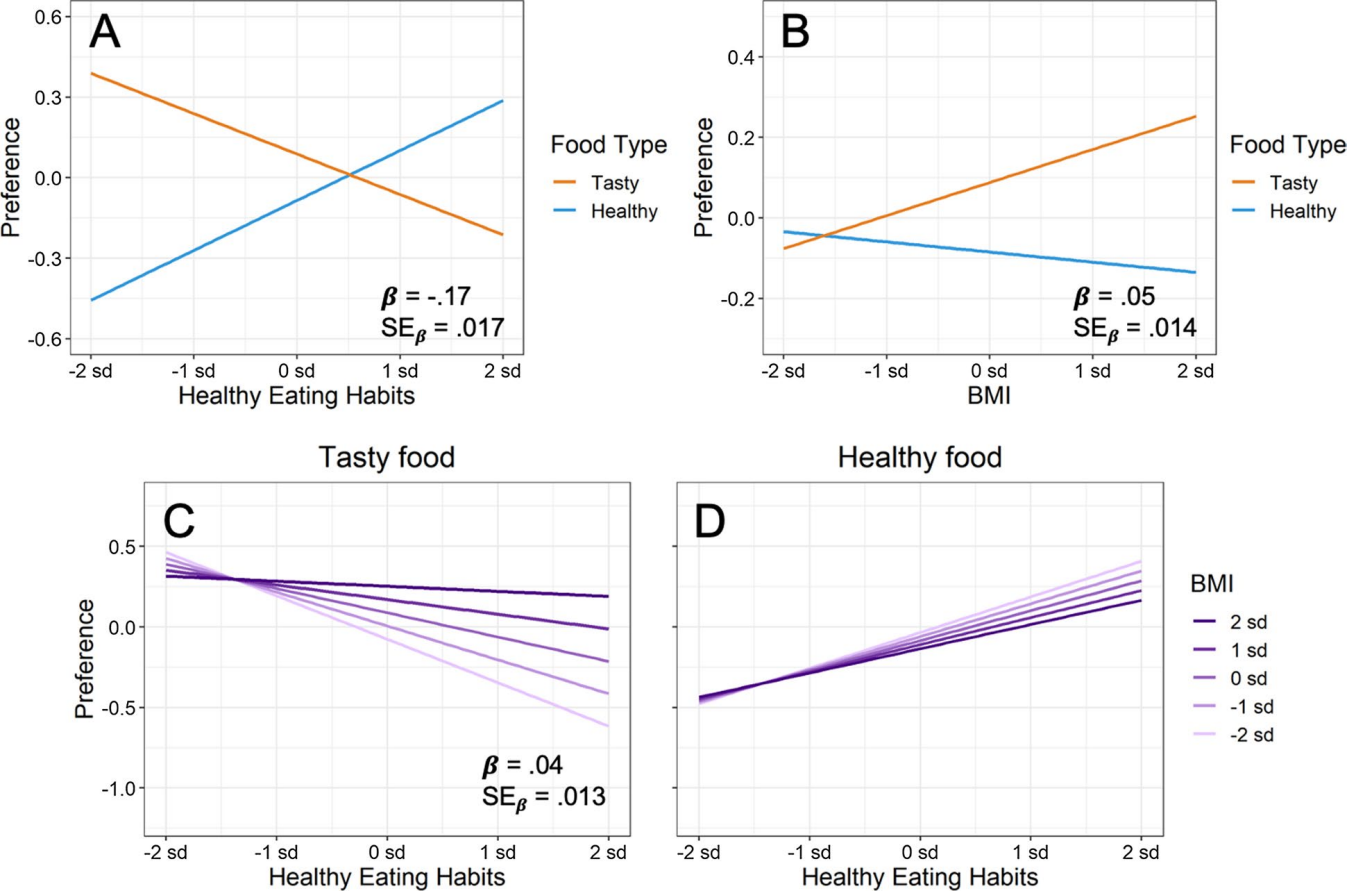
Evidence for the validity of the food preference measure. **A** As participants’ eating habits become increasingly healthy, preferences for healthy foods increased, whereas preferences for tasty foods decreased. **B** Conversely, as participants’ BMI increased, preferences for healthy foods decreased, whereas preferences for tasty foods increased. (C and D) The food type × eating habits × BMI interaction further shows that BMI modulated the food type × eating habits interaction in Panel A. In each panel, a modeled interaction from regression is shown, with all scales plotted in standardized units. From Table 2, the standardized estimated regression coefficient for each interaction is shown (β), together with its standard error to provide a measure of expected variability (SE_β_)

If the food preference measure is valid, it should respond in expected ways to individual differences in healthy eating habits and BMI. Consistent with this prediction, the food preference measure was strongly related to these individual difference measures. As participants’ eating habits became increasingly healthy, preferences for healthy foods increased, whereas preferences for tasty foods decreased (Fig. 3A). Conversely, as participants’ BMI increased, preferences for healthy foods decreased, whereas preferences for tasty foods increased (Fig. 3B). These two interactions indicate that the food preference measure tracked predicted differences in healthy eating habits and BMI, demonstrating its validity.

Table 2 presents the supporting statistical results from regressing food preference onto food type, healthy eating habits, and BMI. As predicted, both the healthy eating habits × food type interaction and the BMI × food type interaction survived maximal testing in Model 2, exhibiting robust estimated regression coefficients (*t* >|1.96|). As a consequence, both interactions generalized across participants and foods here, and are likely to generalize across future participants and foods.

**Table 2:** Mixed-effect regression of food preference on food type (Food), healthy eating habits (Habits), and body mass index (BMI)

DV: food preference	Model 1			Model 2					Model 3	
Predictor	Estimate	SE	*t*	Estimate	SE	*t*	*R*^2^	AIC	∆*R*^2^	AIC
Food type	.09	.038	2.26	.09	.040	2.18	28	76,731	− 7	78,345
Healthy eating habits	.02	.014	1.26							
BMI	.03	.015	1.90							
Food × Habits	− .17	.005	− 32.53	− .17	.017	− 9.96	29	76,532	− 11	79,380
Food × BMI	.05	.005	9.90	.05	.014	3.83	29	76,687	− 7	78,438
Food × Habits × BMI	.04	.006	6.99	.04	.013	3.00	29	76,502	− 8	78,389

Exposure was not included as a factor because the interactions of interest above remained constant across the three exposure conditions (i.e., the regression was performed on all 617 participants). All regressions were performed on standardized measures

The results for Model 3 in Table 2 further indicate that each interaction explained large amounts of unique variance in food preference. Specifically, the healthy eating habits × food type interaction explained 11% unique variance in food preference, and the BMI × food type interaction explained an additional 7%. These large predicted interactions demonstrate that the food preference measure closely tracked important individual differences in eating.

Further validation of the food preference measure comes from the predicted food type × eating habits × BMI interaction in Fig. 3C, D. As just described, Fig. 3A demonstrated that healthy eating habits were associated with decreasing consumption of tasty food and increasing consumption of healthy food. Importantly, however, BMI significantly moderated this interaction. As Fig. 3C illustrates, increasing BMI largely eliminated the decreased preference for tasty foods associated with healthy eating habits. Conversely, Fig. 3D illustrates that increasing BMI diminished the increased preference for healthy foods associated with healthy eating habits, although to a much lesser extent.

As Model 3 in Table 2 illustrates, an additional 8% unique variance in food preference was explained by the food type × eating habits × BMI interaction. Together the three interactions between food type, eating habits, and BMI explained a substantial 26% unique variance in food preference. These strong predicted interactions demonstrate the validity of the food preference measure, showing that it tracks important individual differences related to food consumption that originate outside the laboratory.

**Hypothesis 1**: **Effects** **of** **hedonic** **versus health exposure on food preference**

**Fig. 4:**
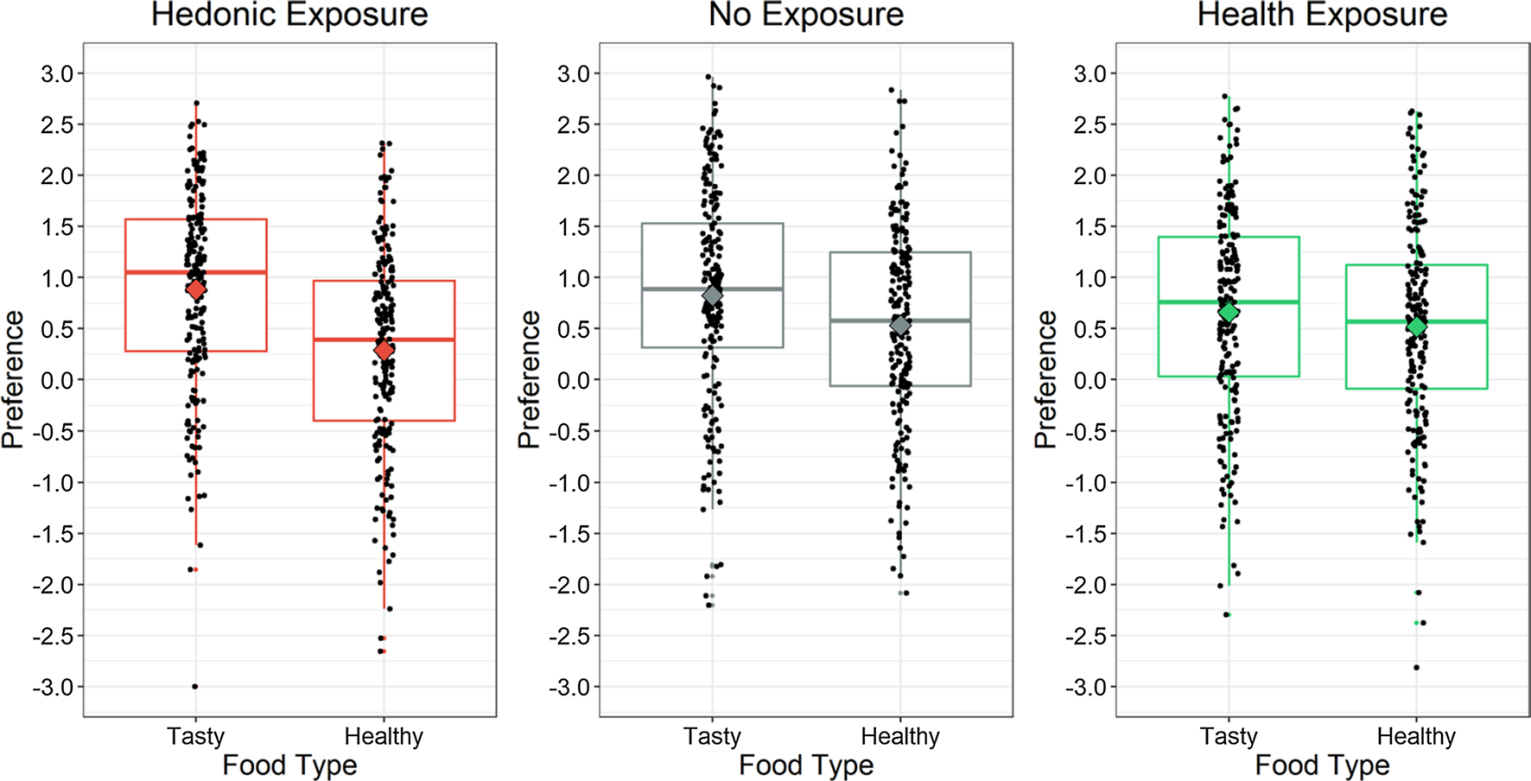
Results for the food preference task. The vertical axis represents responses on the original − 3 to + 3 preference scale. For each exposure group, a diamond represents the mean; a box and whisker plot represents the median and inter-quartile range. Each point represents a participant’s average judgment for either the 24 tasty foods or for the 24 healthy foods

Our central prediction was that incidentally acquired memories of hedonic versus healthy food features would indirectly influence preferences for tasty versus healthy foods a day later. As Fig. 4 illustrates, tasty foods were preferred over healthy foods in both the hedonic and health exposure groups. Notably, however, this preference was much larger following hedonic exposure than following health exposure.

The top section of Table 3 (Hedonic vs. Health Exposure) presents the supporting statistical evidence. As predicted, there was a main effect of food type, with both exposure groups preferring tasty food over healthy food. Most importantly, the predicted food type × exposure interaction survived maximal testing in Model 2, indicating that the preference for tasty foods over healthy foods was stronger following hedonic exposure than following health exposure. Because this interaction survived maximal testing, it generalized across participants and foods here, and is likely to generalize across future participants and foods.

**Table 3:** Mixed-effect regressions of food preference on food type and exposure

DV: food preference	Model 1			Model 2					Model 3	
Contrast/Predictor	Estimate	SE	*t*	Estimate	SE	*t*	*R*^2^	AIC	∆*R*^2^	AIC
*Hedonic vs. Health Exposure*										
Food Type	.09	.040	2.26	.09	.043	2.09	29	51,312	− 11	53,146
Exposure	− .00	.017	− 0.07							
Food Type × Exposure	.06	.006	8.58	.06	.018	3.16	29	51,308	− 11	53,215
*Hedonic vs. No Exposure*										
Food Type	.11	.038	2.83	.11	.041	2.62	28	51,171	− 10	52,883
Exposure	− .02	.017	− 1.32							
Food Type × Exposure	.04	.006	5.72	.04	.017	2.13	28	51,167	− 10	52,909
*Health vs. No Exposure*										
Food Type	.05	.038	1.38							
Exposure	− .02	.018	− 1.20							
Food Type × Exposure	− .02	.006	− 2.81	− .02	.016	− 1.11	28	51,508		

Regressions were performed on standardized measures. For Food Type, tasty foods were coded + 1, and healthy foods were coded − 1. In the Hedonic vs. Health Exposure regression, hedonic exposure was coded + 1, and health exposure was coded − 1. In the Hedonic vs. No Exposure regression, hedonic exposure was coded + 1, and no exposure was coded − 1. In the Health vs. No Exposure regression, health exposure was coded + 1, and no exposure was coded − 1

The results for Model 3 in Table 3 further indicate that the food type × exposure interaction explained a large amount of unique variance in food preference (11%). When hedonic versus health exposure was manipulated between groups of participants, it produced a substantial change in the relative preference of tasty over healthy foods. This result supports our central hypothesis that incidental learning influences food preferences indirectly a day later. Relatively small amounts of exposure to hedonic versus health information can impact food preferences considerably.

**Hypothesis 2**: **Effects of hedonic versus health exposure relative to the no-exposure baseline**

We further predicted that, relative to the no-exposure baseline, only hedonic exposure would produce a group-level effect on food preferences—health exposure would not. Whereas hedonic exposure would increase preferences for tasty foods over healthy foods, health exposure would not decrease this preference (relative to the preference for tasty foods over healthy foods in the no-exposure group). As Fig. 4 illustrates, the difference between hedonic exposure and no-exposure was indeed relatively large, but the difference between health exposure and no-exposure was not.

The second and third sections of Table 3 confirm these observations. For the Hedonic versus No-Exposure contrast, the food type × exposure interaction survived maximal testing in Model 2. For the Health versus No-Exposure contrast, however, the food type × exposure interaction failed maximal testing, reflecting the presence of large individual differences. When random slopes were added for health exposure, food type, and their interaction in Model 2, the exposure × food type interaction became much weaker, indicating that it does not generalize across participants and foods (i.e., *t* = −1.11 <|1.96|). This finding foreshadows the importance of individual differences later when we turn to BMI and healthy eating habits.

As Table 3 further illustrates for the Hedonic versus No-Exposure contrast, the food type × exposure interaction explained a large amount of unique variance in food preference (10%). Relative to the no-exposure baseline, hedonic exposure produced a large increase in the preference of tasty over healthy foods. A relatively small amount of exposure to hedonic information increased the relative preference for tasty foods across foods and participants a day later.

**Hypothesis 3**: **Effects of hedonic and healthy endorsements on food preference**

The endorsement data offer potential insight into the finding for Hypothesis 2 that only hedonic exposure influenced food preferences relative to the no-exposure baseline. Building on that finding, Hypothesis 3 further predicted that preferences for foods would only increase as more hedonic features were endorsed for them—not as more healthy features were endorsed (an exposure × endorsements interaction on food preference). On the one hand, endorsing hedonic features during hedonic exposure should incidentally establish highly accessible affective memories that influence food preferences indirectly a day later. On the other, endorsing healthy features during health exposure should produce less accessible memories that do not influence food preferences generally across participants. As a consequence, food preferences would only be related to the number of hedonic features endorsed, not to the number of healthy features endorsed.

**Fig. 5:**
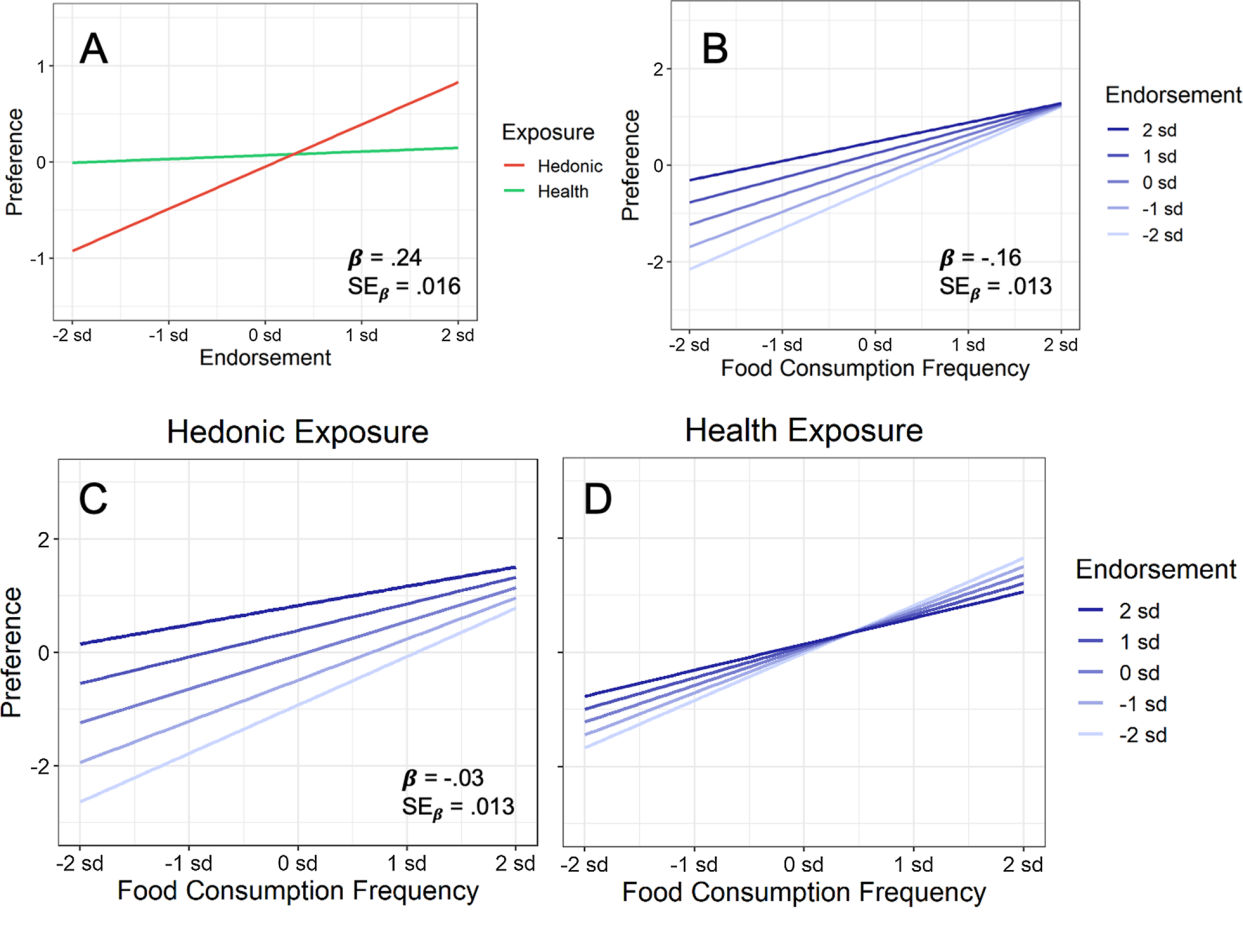
**A** Interaction between exposure condition and endorsement on food preference (for increasing hedonic endorsements in the hedonic exposure condition and for increasing healthy endorsements in the health exposure condition). **B** Consumption frequency × endorsement interaction on food preference. **C**, **D** Endorsement × frequency × exposure interaction on food preference for the hedonic and health exposure conditions individually. In each panel, a modeled interaction from regression is shown, with all scales plotted in standardized units. From Table 4, the standardized estimated regression coefficient for each interaction is shown (*β*), together with its standard error to provide a measure of expected variability (SE_β_)

The results in Fig. 5A confirm these predictions. Food preferences increased with the number of hedonic features endorsed during hedonic exposure but did not increase with the number of healthy features endorsed during health exposure. Although participants clearly discriminated the relative healthiness of tasty versus healthy foods (Fig. 2), their perceptions of healthiness were unrelated to their food preferences (Fig. 5A). Table 4 presents the supporting statistical results, showing that the endorsement × exposure interaction survived maximal testing in Model 2 and explained 4% unique variance in Model 3. Consistent with the results for Hypothesis 2, only hedonic endorsements on day 1 were related to food preferences on day 2.

**Table 4:** Mixed-effect regressions of food preference on predictors that included frequency (Freq) and endorsement (Endorse), along with food type (Food) and exposure (Expo)

DV: food preference	Model 1			Model 2					Model 3	
Predictor	Estimate	SE	*t*	Estimate	SE	*t*	*R*^2^	AIC	∆*R*^2^	AIC
Frequency	.62	.010	61.80	.63	.024	26.82	64	39,816	− 17	44,712
Endorsement	.24	.009	25.83	.27	.016	16.27	61	40,424	− 4	41,885
Food Type	.07	.026	2.52	.08	.028	2.95	59	40,450	− 2	41,236
Exposure	− .06	.018	− 3.30	− .06	.020	− 2.85	59	41,135	− 1	41,241
Frequency × Endorsement	− .11	.009	− 12.36	− .16	.013	− 12.26	67	38,838	− 9	41,382
Frequency × Exposure	− .02	.010	− 2.41	− .02	.015	− 1.28	65	39,686		
Endorsement × Exposure	.20	.009	21.52	.24	.016	14.85	61	40,372	− 4	41,688
Food × Exposure	− .07	.010	− 7.19	− .09	.017	− 5.55	60	40,343	− 2	41,282
Freq × Endorse × Food	− .02	.009	− 2.49	− .04	.012	− 2.83	68	38,528	− 10	41,236
Freq × Endorse × Expo	− .02	.009	− 1.96	− .03	.011	− 2.74	67	38,777	− 9	41,234
Freq × Food × Expo	.07	.010	6.84	.08	.012	6.68	67	38,927	− 9	41,277
Endorse × Food × Expo	− .03	.010	− 3.23	− .03	.013	− 2.33	62	40,098	− 4	41,241

Regressions were performed on standardized measures. For Food Type, tasty foods were coded + 1, and healthy foods were coded − 1. For Exposure, hedonic exposure was coded + 1, and health exposure was coded − 1

**Hypotheses 4**: **Effects of consumption frequency on food preference**

We predicted that eating habits—as reflected in a participant’s reported consumption frequency for each food—would heavily influence their food preferences. As a participant consumed a food more often, their preference for it would increase.

Figure 5B plots the results, and Table 4 presents the statistical analysis. As Fig. 5B illustrates, food preference increased substantially as consumption frequency increased. As Table 4 documents, consumption frequency explained more unique variance in food preference than any other predictor in the experiment (17%), with a standardized regression coefficient of 0.62. As hypothesized, eating habits strongly predicted food preferences.

Because eating habits have much more strength in memory than information acquired via brief exposure, Hypothesis 4 further predicted that eating habits should dominate food preferences relative to endorsements. As a consequence, the endorsement effect just reported in Fig. 5A for Hypothesis 3 was predicted to become minimal at high levels of consumption frequency (a frequency × endorsements interaction on food preference). As Fig. 5B and Table 4 illustrate, consumption frequency did indeed interact with endorsements, explaining 9% of the variance in food preference. Consistent with our prediction, the effect of endorsements was weakest at the highest levels of consumption frequency. Whereas endorsements had large effects on food preferences for foods consumed occasionally, they had relatively little effect for foods consumed frequently. Together, frequency, endorsements, and their interaction explained a total 30% unique variance in food preference.

As we also saw in Fig. 5A for Hypothesis 3, the endorsement effect only occurred for hedonic exposure. It follows that the attenuating effect of consumption frequency on endorsements should therefore occur primarily for hedonic exposure and not for health exposure (an exposure × frequency × endorsements interaction on food preference). As Fig. 5C, D illustrate, frequency and endorsements did indeed interact with exposure. In Table 4, the exposure × frequency × endorsements interaction survived maximal testing in Model 2 and explained an additional 9% unique variance in Model 3. Whereas increasing endorsements and increasing consumption frequency both increased food preference following hedonic exposure, only increasing consumption frequency increased food preference following health exposure. Again, increasing healthy endorsements had no overall effect on food preference (illustrated previously in Fig. 5A).

Nevertheless, the frequency × endorsements interaction exhibited a common property across both the hedonic and health exposure conditions: As hedonic and healthy endorsements increased, they each attenuated the effect of consumption frequency on food preference (Figs. 5C, D). In both cases, increasing endorsements “flattened out” the strong effect of consumption frequency.

Importantly, however, hedonic endorsements attenuated the frequency effect much more than did healthy endorsements (i.e., the frequency effect became much flatter with increasing hedonic endorsements in Fig. 5C than with increasing healthy endorsements in Fig. 5D). Additionally, food preference increased for each increasing level of hedonic endorsements in Fig. 5C, but not with each increasing level of healthy endorsements in Fig. 5D. This latter effect essentially reflects the endorsements × exposure interaction presented earlier in Fig. 5A, where food preference only increased with hedonic endorsements but not with healthy endorsements.

Finally, increasing hedonic endorsements were associated with higher food preferences across all levels of consumption frequency, from low to high, with the increase becoming smaller as frequency increased. Increasing healthy endorsements, however, behaved differently. At low levels of frequency, increasing healthy endorsements increased food preferences, but at high levels, increasing healthy endorsements decreased food preferences. The latter effect could reflect the fact that foods high in healthy features are also low in hedonic features, leading to lower preferences.

**Hypothesis 5**: **BMI modulates the effect of health exposure.**

As reported for Hypothesis 2, we found that health exposure did not have a general effect across participants relative to the no-exposure baseline (Fig. 4, Table 3). We only expected that health exposure would influence food preferences for individuals who are likely to be concerned with their body weight. To assess this possibility, we assessed relations between health exposure and individual difference measures for BMI, healthy eating habits, and dietary restraint. We only report analyses for BMI and healthy eating habits because: (a) BMI and healthy eating habits were relatively unrelated (*r* = − 0.09), (b) restraint correlated with healthy eating habits (*r* = 0.52), and (c) restraint behaved much like healthy eating habits in the analyses to follow but showed weaker effects. Similar to healthy eating habits, restraint also correlated -0.09 with BMI.

**Fig. 6:**
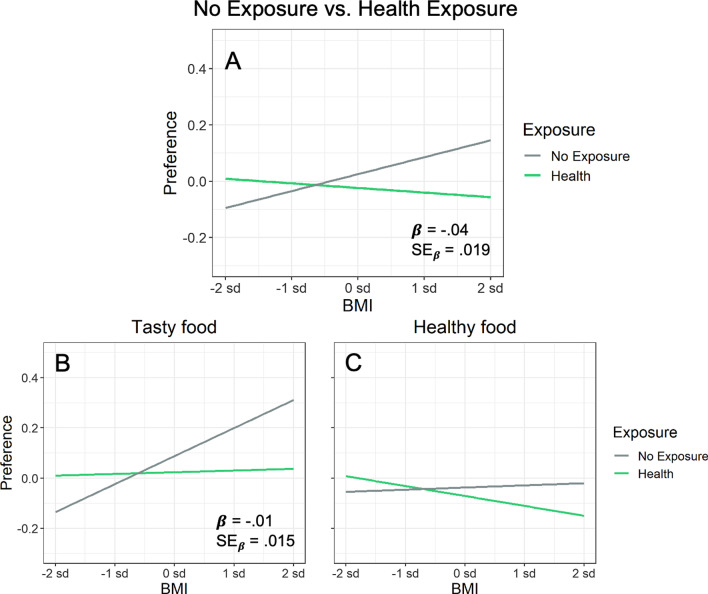
**A** Exposure × BMI interaction on food preference. **B**, **C** Interaction individually for tasty and healthy foods, respectively (illustrating the *lack* of an exposure × BMI × food type interaction). In each panel, a modeled interaction from regression is shown, with all scales plotted in standardized units. From Table 5, the standardized estimated regression coefficient for each interaction is shown (*β*), together with its standard error to provide a measure of expected variability (SE_β_)

To assess whether BMI and eating habits modulated the effect of health exposure relative to the no-exposure baseline, we assessed these individual difference measures in an analysis that contrasted health exposure with no-exposure. Figure 6 plots the relevant results, and the top half of Table 5 presents the statistical analysis. As Fig. 6A illustrates, BMI modulated the effect of health exposure. For the no-exposure condition in Fig. 6A, overall food preference increased with BMI, as normally expected. Following health exposure, however, this tendency disappeared and even reversed, such that overall food preference actually decreased slightly with BMI.

**Table 5:** Mixed-effect regressions of food preference on predictors that included healthy eating habits (Habits) and BMI, along with food type (Food) and exposure (Expo)

DV: food preference	Model 1			Model 2					Model 3	
Predictor	Estimate	SE	*t*	Estimate	SE	*t*	*R*^2^	AIC	∆*R*^2^	AIC
*Health Exposure vs. No Exposure*										
Food Type	.06	.038	1.45							
Expo sure	− .03	.018	− 1.38							
Healthy eating habits	.01	.018	0.74							
BMI	.02	.019	1.15							
Food × Habits	− .18	.006	− 27.81	− .18	.018	− 9.94	29	51,259	− 10	53,000
Food × BMI	.04	.007	5.51	.04	.016	2.33	28	51,335	− 6	52,272
Exposure × Habits	− .04	.018	− 2.06	− .04	.018	− 2.03	23	52,163	− 1	52,246
Exposure × BMI	− .04	.019	− 2.01	− .04	.019	− 2.01	22	52,234	− 1	52,245
Food × Expo × BMI	− .01	.007	− 2.10	− .01	.015	− 0.98	28	51,341		
Food × Habits × BMI	.04	.007	6.19	.04	.015	2.74	29	51,242	− 7	52,280
Expo × Habits × BMI	− .04	.019	− 2.21	− .04	.019	− 2.20	23	52,157	− 1	52,246
Food × Expo × Hab × BMI	− .01	.007	− 2.09	− .01	.014	− 0.96	29	51,249		
*Hedonic Exposure vs. No Exposure*										
Food Type	.11	.038	2.96	.11	.040	2.80	28	51,030	− 6	52,032
Exposure	− .02	.017	− 1.35							
Healthy eating habits	.04	.017	2.46	.04	.022	1.91	23	51,885		
BMI	.04	.018	2.38	.04	.020	2.24	22	52,010	0	52,030
Food × Exposure	.05	.006	7.28	.05	.015	3.14	28	51,025	− 7	52,077
Food × Habits	− .16	.006	− 25.64	− .16	.020	− 8.27	29	50,867	− 10	52,671
Food × BMI	.08	.007	11.07	.08	.017	4.57	29	51,007	− 7	52,146
Habits × BMI	.04	.019	2.27	.04	.020	2.23	23	51,859	− 1	52,029
Food × Expo × Habits	.02	.006	2.82	.02	.014	1.26	29	50,862		
Food × Expo × BMI	.03	.007	3.76	.03	.015	1.69	29	51,011		
Food × Habits × BMI	.05	.007	6.52	.05	.016	2.92	30	50,852	− 8	52,067

Regressions were performed on standardized measures. For Food Type, tasty foods were coded + 1, and healthy foods were coded − 1. In the Hedonic vs. No Exposure regression, hedonic exposure was coded + 1, and no exposure was coded − 1. In the Health vs. No Exposure regression, health exposure was coded + 1, and no exposure was coded − 1

Although the Exposure × BMI interaction survived maximal testing in Model 2 (Table 5), the food type × exposure × BMI interaction did not, indicating that the decrease in food preference with BMI occurred for *both* tasty and healthy foods. Figure 6B, C illustrate this common predicted decrease across the two food types. This pattern indicates that health exposure diminished food preference across both tasty and healthy foods, suggesting that dieting goals became engaged and tempered overall interest in food.

**Fig. 7:**
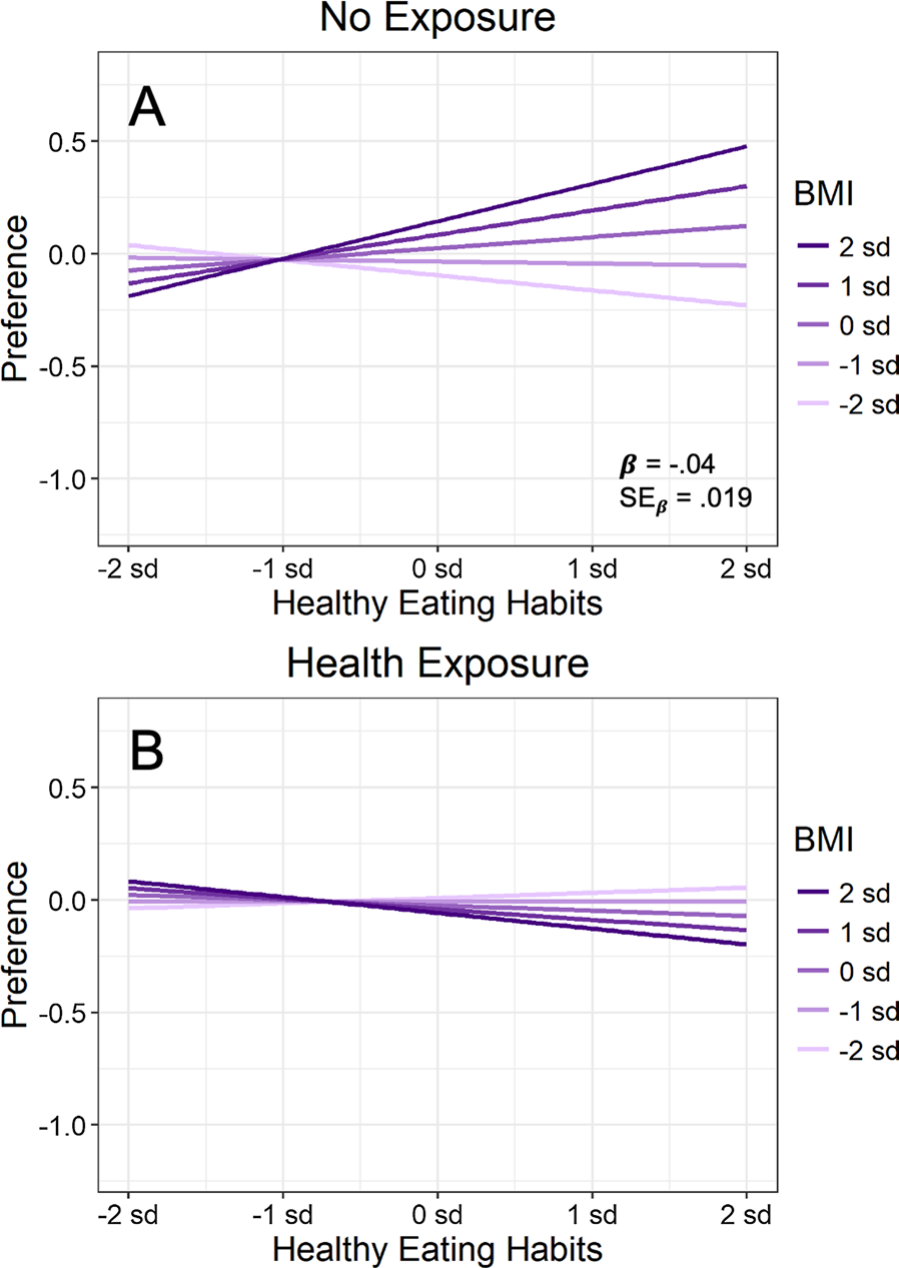
**A**, **B** Exposure × healthy eating habits × BMI interaction on food preference for the no-exposure and health exposure groups, respectively (combined across tasty and healthy foods). In each panel, a modeled interaction from regression is shown, with all scales plotted in standardized units. From Table 5, the standardized estimated regression coefficient for the interaction is shown (*β*), together with its standard error to provide a measure of expected variability (SE_β_)

Figure 7 presents a non-preregistered interaction between BMI and health exposure established in discovery mode (the top half of Table 5 provides the statistical details). In the no-exposure group, BMI interacted with healthy eating habits for preferences of tasty and healthy foods combined (Fig. 7A). Specifically, at low BMI, overall food preference decreased as healthy eating habits increased. Conversely, at high BMI, overall preference increased as eating habits became healthier. This interaction in the no-exposure group illustrates that high BMI counteracted the benefits of healthy eating habits on overall food preference. As Fig. 7B illustrates, however, health exposure completely eliminated this effect of BMI. Health exposure reversed the relation of BMI to overall preference, such that the lowest overall preference levels occurred for individuals high in both BMI and healthy eating habits.

Finally, the lower half of Table 5 further confirms the generalizability of the hedonic exposure effect reported earlier for Hypothesis 2. As found earlier, the effect of hedonic exposure relative to the no-exposure baseline survived maximal testing in Model 2, demonstrating that it generalizes across foods and participants (Fig. 4, Table 3). The lower half of Table 5 further illustrates that the hedonic exposure effect generalizes across individual differences in BMI and healthy eating habits. Specifically, in the contrast between hedonic exposure and no-exposure, no interaction of hedonic exposure with either BMI or healthy eating habits survived maximal testing in Model 2. In other words, individual differences in BMI and healthy eating habits did not modulate the effect of hedonic exposure. Again, hedonic exposure appears to have a robust effect that generalizes broadly.

### Assessing demand

On the questions that assessed experimenter demand, no evidence of demand was observed. When participants were asked whether their responses reflected personal assessments, their median response was 6 (all responses were made on 0 to 6 slider scales). When asked whether they responded naturally and intuitively without a lot of deliberate thought, their median response was 5.7. Conversely, when participants were asked whether they responded in a way that the experimenters wanted to hear, their median response was 0. When asked whether they thought there were correct answers to the survey questions, their median response was 0.

Most importantly, responses to these four questions did not differ between the two exposure conditions and the no-exposure condition. If demand had been operating in the exposure conditions, we should have seen lower responses for both conditions on the first two questions above relative to the no-exposure condition, and higher responses on the last two questions above. In other words, the initial exposure session should have created demand that was not observed in the no-exposure condition. In linear regressions that contrasted each type of exposure with no-exposure, no hint of an exposure effect appeared, thus providing no evidence of demand (median estimated regression coefficient = − 0.02, median *t* = − 0.49).

## Discussion

### Summary of results

We introduced a novel paradigm for assessing two classic memory processes in exposure to food information: incidental learning and indirect memory. To minimize demand during the food preference phase, we used a comprehensive cover story to obscure the relation between the incidental learning of food information initially and food preference judgments later. As a consequence, participants had no reason to intentionally learn or deliberately remember hedonic or health information from the exposure phase, such that any effects of this information during the preference phase occurred indirectly.

#### Hedonic versus health exposure

Incidental exposure to hedonic versus healthy food features affected food preferences one day later. Just two exposures to food features changed relative preferences for tasty versus healthy foods, with the interaction between exposure and food type explaining 11% unique variance in food preferences. Assuming that this manipulation falls on the early part of a dose–response curve, many more exposures could have still larger effects. It further follows that brief exposure to food information can be expected to affect food preferences for at least one day.

#### Assessing hedonic and health exposure against a no-exposure baseline

When hedonic and health exposure were each compared to a no-exposure baseline, only a strong group-level effect of hedonic exposure emerged. Following exposure to hedonic food features, overall food preference increased for tasty foods, relative to the no-exposure baseline. Because the effect of hedonic exposure survived maximal testing in our analysis procedure, it not only generalizes across participants and foods here but is also likely to generalize across future participants and foods. Further evidence for the generalizability of hedonic exposure comes from its lack of interaction with BMI and healthy eating habits. Hedonic exposure affected individuals across a broad range of individual differences associated with eating.

We speculate that the general effect of hedonic exposure reflects basic affective mechanisms associated with pleasure and reward that operate outside conscious awareness and self-regulation (e.g., Rolls, 2015). As participants endorsed hedonic features during hedonic exposure, they may have simulated the experience of “liking” foods, perhaps followed by “wanting” them (Berridge, 1996; Berridge et al., 2009). As neuroimaging research shows, focusing attention on the hedonic qualities of foods activates brain areas associated with hedonic enjoyment and reward (e.g., Chen et al., 2016a, 2016b; Pelchat et al., 2004; Siep et al., 2012). Imagining the pleasure of eating may occur naturally across most individuals, leaving behind robust memories that later become active indirectly to influence food preferences.

#### Individual differences in the effect of health exposure

Unlike hedonic exposure, health exposure did not produce a general effect across individuals in food preferences. Although participants in the health exposure condition clearly perceived a large difference in the healthiness of tasty versus healthy foods during exposure, these perceptions did not affect group-level preferences.

Importantly, however, health exposure *did* affect food preferences for individuals high in BMI, causing them to lower their overall food preferences for *both* tasty and healthy foods. During health exposure, healthy features of food may have become salient and important for high-BMI individuals, establishing robust memories of food healthiness incidentally. When these individuals performed the food preference task the next day, these robust memories were highly available and became active to affect their preferences. As a consequence, high-BMI participants adopted a restrained perspective on food consumption that reduced their overall preferences for both tasty and healthy foods (consistent with reducing overall calorie intake).

#### Impact of eating habits

The prior frequency of consuming foods explained more variance in food preference than any other factor, demonstrating the powerful effect of habits on behavior (Marteau et al., 2012; Ouellette & Wood, 1998; Verplanken, 2018). People have a strong tendency to choose foods for a meal that they typically eat for it.

As further predicted, exposure to hedonic and healthy food information interacted with consumption frequency. In general, exposure to food information attenuated the effect of frequency, weakening its relation to food preference. As both hedonic and healthy endorsements increased, they weakened the frequency effect. Importantly, however, hedonic endorsements attenuated the frequency effect much more than did healthy endorsements. Healthy endorsements had relatively little impact on the consumption frequency effect, further illustrating the relatively weak effects of health exposure.

### Relations to previous research

To our knowledge, the research presented here is the first to assess whether incidentally acquired memories of hedonic versus healthy food features affect food preferences indirectly. Related experimental work in two other areas, however, complements our work.

#### Cognitive training

One prominent line of research implements cognitive training on eating and other health behaviors, and then assesses the impact of this training on behavior change (for reviews, see Cristea et al., 2016; Jones et al., 2018; Kakoschke et al., 2017; Stice et al., 2016). Most typically, these paradigms implement the training of *attention* (attending to healthy stimuli and avoiding unhealthy stimuli), *inhibition* (inhibiting responses to unhealthy stimuli), and *approach-avoidance responses* (approaching healthy stimuli and avoiding unhealthy stimuli). In all cases, the focus is on training some form of action, ranging from attention to motoric movements.

An emerging theme in this literature is the importance of making health consequences salient. Research on inhibition and approach-avoidance training increasingly concludes, for example, that the active ingredient in such training is not action related to food per se (e.g., inhibition, approach, avoidance), but the inferred *consequences* of performing these actions, such as good versus poor health outcomes (Chen et al., 2016a, b; Eder & Hommel, 2013; Stice et al., 2016; Van Dessel et al., 2018).

Whereas cognitive training changes actions related to eating, our paradigm establishes incidentally acquired food memories that become active indirectly to affect food preferences. A commonality across both approaches is a focus on the consequences of eating. Similar to cognitive training, our incidental learning procedure establishes the hedonic or healthy consequences of consuming specific foods. An important difference, however, is that our paradigm induces the incidental processing of hedonic and health consequences *in the absence of explicitly training action*. Simply strengthening the consequences of eating in memory affected food preferences, even when actions were not trained.

#### Health priming

A second prominent line of research places cues in an individual’s immediate environment to prime healthy eating goals, which in turn aim to induce healthy eating. Field studies, for example, have used a wide variety of environmental cues to effectively influence eating goals, where these cues include posters, slogans, menus, recipes, and screensavers (Berger & Fitzsimons, 2008; Brunner & Siegrist, 2012; Papies & Hamstra, 2010; Papies & Veling, 2013; Papies et al., 2014; Stöckli et al., 2016). As these studies show, using environmental cues to prime health goals can induce healthier eating preferences, choices, and consumption.

Importantly, health priming is most successful in individuals who have established healthy eating goals during their previous eating behavior (Buckland et al., 2018; Papies, 2016a, b). When individuals have not previously established such goals, health primes typically have little if any effect on food preferences. Consistent with our finding that health exposure only influenced food preferences in high-BMI individuals, having a healthy eating goal already in place is important for a health priming intervention to work. Health primes only have an effect when a well-established healthy eating goal is available to prime.

Well-controlled laboratory experiments similarly find that explicitly priming healthy versus hedonic eating goals can have immediate effects on food preference (e.g., Boswell et al., 2018; Hollands & Marteau, 2016; Hollands et al., 2011; Young & Fazio, 2013). Effective priming procedures in the laboratory include asking participants to evaluate foods for their healthiness versus tastiness, or asking participants to associate foods with images of healthy versus unhealthy eating outcomes. Similar to environmental cues in field studies, these laboratory procedures prime eating goals that influence subsequent food preferences. Also similar to field studies, individual differences in BMI and healthy eating habits moderate these priming effects.

Our paradigm differs from health prime paradigms because *it does not include any cues in the immediate environment that induce priming*. In our experiment, participants simply evaluated individual foods with no health primes present. Additionally, our key manipulation occurred one day earlier during the exposure phase, when participants acquired either healthy or hedonic food memories of foods incidentally. Of interest was whether these memories became active indirectly as the foods were encountered again a day later. Rather than assessing health priming, our paradigm assessed indirect activation of incidentally acquired information. In our paradigm, no cues were present when food preferences were evaluated that could differentially prime healthy or hedonic eating goals.

An important implication of health priming research is that priming a health goal can have considerable impact. When a health prime is present in an individual’s immediate environment, it can activate healthy eating goals that induce healthy eating behavior. When a health prime is not present, however, the indirect activation of incidentally acquired food information is likely to dominate food preferences instead. Our findings provide insight into what happens under these conditions: Hedonic memories are more likely to influence food preferences than healthy memories, except for high-BMI individuals. Although the priming of health goals offers an important mechanism for influencing immediate food preferences, it is a different mechanism than the indirect activation of incidental memories.

### Assessing exposure effects on consumption

Eating is *not* a simple one-act event of consumption, but is a “multifaceted, contextual, dynamic, multilevel, integrated, and diverse” activity that unfolds across time, space, and culture (Sobal et al., 2014, p. 6). From this perspective, it is important to understand the preliminary processes associated with consumption—not just consumption itself—including the eating preferences and intentions that arise during meal planning and shopping. Interventions can target not only final acts of food consumption, but also preliminary processes that play central roles in producing these acts. In Chile, for example, increasing research demonstrates that Chilean food labeling policies affect preliminary processes that precede food purchases, long before the consumption that eventually follows (e.g., Durán Agúero et al., 2020; Taillie et al., 2020). Our work similarly demonstrates that exposure to food information affects preliminary processes associated with food consumption. Exposure to hedonic and healthy food features changed preferences one day later for consuming tasty versus healthy foods at specific meals.

Our approach further lends itself to assessing the effects of hedonic and health exposure on consumption itself. Instead of assessing food preferences one day following exposure, one could assess eating behavior. Does hedonic and/or health exposure affect the relative amounts of tasty and healthy food consumed? Because of problems associated with measuring consumption in the laboratory (e.g., Best et al., 2018; Robinson et al., 2018), our preference would be to assess consumption in people’s normal everyday eating situations, using interview techniques shown to be highly effective (e.g., the Automated Multiple-Pass Method; Subar et al., 2015; Thompson & Subar, 2017), or using emerging mobile technologies.

Much remains to be learned about the pathway from exposure to preference to intention to consumption, with this pathway likely differing for hedonic versus health exposure. Process models are needed that combine current eating habits with information acquired through exposure to produce preferences and intentions on specific occasions. These accounts must further combine preferences and intentions with other contextual factors that determine consumption in immediate eating situations. Our work sheds light on one part of this overall pathway.

### Developing effective interventions

A sobering finding from our experiments is that exposure to hedonic food features increased the preference for tasty foods over healthy foods across broad individual differences in BMI and healthy eating habits. Another sobering finding is that exposure to healthy food features had little impact on making food preferences healthier, except for high-BMI individuals. These findings offer insight into the problems of overweight and obesity in the obesogenic food environment (e.g., Marteau et al., 2012; Norman et al., 2016; Papies, 2016a, 2017). The effectiveness of exposing people to hedonic food features, coupled with the relative ineffectiveness of exposing them to healthy features, offers one reason why maintaining a healthy body weight can be so difficult.

Given the strong hedonic orientation that people take to eating—as evidenced by the robust effects of hedonic exposure here—one approach to increasing the consumption of healthy foods is to make the experience of consuming these foods more hedonic. Increasing work does indeed demonstrate that bringing out the hedonic qualities of healthy foods can increase their consumption (e.g., Papies et al., 2020; Robinson et al., 2012; Turnwald & Crum, 2019).

A contrasting approach is to minimize contributions of hedonic processing and instead draw attention to the importance of long-term health consequences. As we just saw, priming health consequences increases the immediate likelihood of healthy behavior. As also noted, however, when health primes are not present, incidental memories of hedonic experiences are likely to dominate preference, leading to unhealthy behavior. If so, then a key issue becomes how to best activate healthy eating goals in situations where people may instead be more naturally inclined to adopt hedonic ones.

One possibility is designing shopping and eating environments to prime healthy eating behavior (e.g., Hollands et al., 2017; Papies, 2017; Pechey et al., 2020; Rosenblatt et al., 2018). To change eating behavior, change the eating environment (Marteau et al., 2012). By manipulating the availability, positioning, and properties of foods in food choice situations, social policy can shift food preferences away from unhealthy foods towards healthy foods. Certainly, it is also important to continue pursuing internal forms of behavior change, but evidence increasingly implicates the critical importance of externally encouraging healthy preferences while discouraging unhealthy ones (Cadario & Chandon, 2019).

## Conclusion

We have shown that brief exposure to food information establishes memories incidentally that become active indirectly a day later to influence preferences for tasty vs. healthy foods. Relative to a no-exposure baseline, exposure to hedonic food information increased the preference for tasty foods over healthy foods, an effect that generalized across foods, participants, BMI, and healthy eating habits. In contrast, exposure to healthy food information did not have a robust effect, only influencing food preferences in high-BMI individuals. In the absence of environmental cues that prime health goals, incidentally acquired memories of food information are likely to influence eating preferences indirectly.

## Supplementary Information

**Additional file 1.** Supplementary Material (SM).

## Data Availability

All data files, analysis scripts, and materials for all experiments can be found at the Open Science Foundation’s online repository. The materials for all experiments can be found here, https://osf.io/ys4q2/. All data files and analysis scripts for the pilot experiment, the combined experiment, and Parts A and B of the combined experiment can be found here, https://osf.io/s5u3p/. A supplementary document that describes the methods and results of the pilot experiment can be found here, https://osf.io/y2zpk/, along with another supplementary document that presents the theoretical framework motivating the research.

## Footnotes

1 We considered a variety of active baselines as well, but each was associated with a bias towards hedonic or healthy foods (e.g., simple exposure to food pictures without the endorsement task is likely to induce hedonic processing; Papies et al., 2012; Papies et al., 2015). Furthermore, by contrasting hedonic exposure with health exposure, we compared two conditions that contained the same amount of stimulus presentation and processing across two days. If neither type of exposure had an effect (or if they both simply produced an overall fluency effect relative to the no-exposure baseline), then there should have been no differences between them. To the contrary, we observed substantial differences between the two exposure conditions across experiments, each producing a unique pattern of results. Thus, when one exposure group was treated as an active control for the other, large predicted differences occurred, demonstrating that both exposures have unique effects. Additionally, the absence of an overall exposure effect rules out the possibility that the exposure conditions produced a simple fluency effect relative to the no-exposure baseline. Various results suggest instead that food features processed during the endorsement task were responsible for the specific exposure effects observed. Later, Hypothesis 1 captures this contrast between two equally matched exposure conditions, with each exposure condition serving as an active control for the other. Additionally, Hypothesis 2 captures the contrast of a single exposure condition to a passive no-exposure baseline, as described in the text. Thus, both active and passive baseline were addressed and captured in the results reported.

2 We assess BMI here because it remains a widely used and accepted measure in current eating research. Nevertheless, it is important to note that BMI is limited in various ways. For example, high BMI does not always indicate poor health (e.g., body builders), nor consumption of unhealthy food (e.g., people who eat too much healthy food). Additionally, BMI can inadvertently promote fat-shaming and negative stereotypes towards populations associated with greater body weight (e.g., low SES, various racial and ethnic groups). Notwithstanding such limitations, BMI nevertheless remains strongly associated with unhealthy eating behavior, body fat, and poor health outcomes in eating research. Consistent with these widespread results, we found that BMI strongly predicted preferences for tasty and healthy foods (Fig. 3, Table 2). We also found that BMI predicted effects of health exposure (Figs. 6 and 7, Table 5), as found widely in the literature. Throughout our experiment, BMI behaved as expected, providing a valid statistical measure of unhealthy eating behavior.

3 Here, we list preregistered hypotheses for the combined experiment that are not addressed in the main text. Most often, these hypotheses were pre-registered for the combined experiment only because they were significant in Part A, or seemed like they would become significant with the increased power that resulted from combining Parts A and B. Otherwise, these hypotheses were typically not of interest. All details can be found on OSF in a Preregistrations Summary document (https://osf.io/y2zpk/).

- We predicted an exposure × frequency × food type interaction on food preference. As Table 4 in the main text shows, this interaction did indeed occur, significant for Model 2. We only preregistered this hypothesis because it was significant in Part A, not because it was of interest. Indeed, we’re not sure how to interpret it.
- We originally predicted a four-way interaction between exposure × food type × BMI × healthy eating habits because it appeared significant in Part A. When, however, the lmer package that produced it was updated at one point, this interaction was no longer significant. By then, however, we had pre-registered the interaction for Part B and the combined experiment, where it was also not significant. Again, we only pre-registered it for the combined experiment it because it initially appeared present in Part A.
- We originally predicted a food type × exposure × healthy eating habits interaction, first, when hedonic exposure was contrasted with no-exposure, and second, when health exposure was contrasted with no-exposure. Because these interactions appeared present in Part A to varying extents, we thought that they would likely emerge with the greater power of the combined experiment. They did not. Again, we only preregistered this hypothesis because this interaction appeared present in Part A, not because it was of interest or easily interpretable.
- When comparing hedonic exposure to no-exposure, we originally predicted that hedonic exposure would interact significantly with BMI. This interaction did not reach significance in Part A. Because, however, a hint of it appeared in Part A, we preregistered it for the combined experiment. Contrary to our speculation, this interaction was again not significant in either Part B nor in the combined experiment. Because it didn’t actually reach significance in Part A, we probably should not have preregistered it in the first place.
- Because we had performed successful mediation analyses for Part A, we preregistered the same mediation analyses for Part B and the combined experiment. Following these preregistrations, we decided that our designs did not meet the assumptions for mediation analysis, and so removed these results from the current article. We note, however, that all mediation analyses supported all preregistered hypotheses related to them (complete analyses can be found the Preregistrations Summary document at https://osf.io/y2zpk/). We further note that other results that we do report in the main article capture the critical components of the mediation analyses.

## Declarations

### Preregistrations

Preregistration for the Combined Experiment reported here, along with preregistrations for Parts A and B of it, can be found at the Open Science Foundation’s online repository. The preregistration for the combined experiment reported in this article can be found here, https://osf.io/aes79/. The preregistration for Part A of the combined experiment can be found here, https://osf.io/re5mw/. The preregistration for Part B of the combined experiment can be found here, https://osf.io/aes79/. Finally, a summary document that integrates the three preregistrations for all studies, together with the results that bear on each prediction, can be found here, https://osf.io/y2zpk/.
