# Supplementary material for: Incidental exposure to hedonic and healthy food features affects food preferences one day later
Source: Cogn Res Princ Implic. 2021 Dec 11;6:78. doi: 10.1186/s41235-021-00338-6 (PMC8665956; doi:10.1186/s41235-021-00338-6)
Supplement: Supplementary file 1 — Additional file 1. Supplementary Material (SM). [file 41235_2021_338_MOESM1_ESM.pdf]

Supplemental Material (SM)

Incidental exposure to hedonic and healthy food features affects food preferences one day later

Léo Dutriaux[1]

Esther K. Papies[1]

Jennifer Fallon[1]

Leonel Garcia-Marques[2,3]

Lawrence W. Barsalou[1]

[1] School of Psychology and Neuroscience, University of Glasgow
[2] CICPsi Research Center for Psychological Science, University of Lisbon
[3] School of Psychology, University of Lisbon

Contents

Power analysis 2

Images and descriptions for the foods and gifts 3
    Figures SM-1, SM-2, and SM-3

Average endorsement, preference, and frequency for the 48 foods 7
    Tables SM-1 and SM-2

Figures and tables for Parts A and B of the combined experiment reported in the article 10
    Figures SM-4 through SM-9
    Tables SM-3 through SM-7

Power analysis

The power analysis for the experiment reported in the article was based on the results of the Pilot experiment. A document on our OSF website provides a complete account of the Pilot's methods and results (https://osf.io/ys4q2/).

In the Pilot experiment, we observed strong effects for all predictions, using a sample size of 18 in the hedonic exposure group and 21 in the health exposure group. To assess the Pilot's power, we performed a retrospective power analysis for the critical exposure by food type interaction (i.e., a standardized coefficient of .18). Specifically, we wrote an R script that performed a power analysis using Monte Carlo simulation. The script simulated the critical 48 trials in the preference phase of the Pilot experiment for each simulated participant, using the mixed-effects model from its analysis. For each simulated trial, the model included the estimated regression coefficients from the Pilot for the fixed effects of exposure, food type, and exposure X food type. Additionally, the model included randomly sampled values for the following variance components: residuals (one for each trial in a simulation), random intercepts for participants and foods (one for each participant and food in a simulation), random slopes for the exposure and food type manipulations (again one for each participant and food in a simulation). The values for each randomly sampled variance component were drawn from a normal distribution with a mean of 0 and the same standard deviation as the variance component in the original model. Because participants were nested in exposure and because foods were nested in food type, random slopes for the exposure by food type interaction did not exist (nor random slopes of participants for exposure, or random slopes of foods for food type). Correlations between random effects were not included, given the complexities associated with simulating them, and the relatively small impact they have on detecting significant effects (Barr, Levy, Scheepers, & Tily, 2013). We ran 10,000 simulations with a sample size of 19 (given the Pilot experiment had sample sizes of 18 and 21 in its two conditions), and found that the Pilot had power of 83% to detect the interaction.

Because we now wanted to assess each exposure condition (hedonic and healthy) against a neutral baseline, we predicted that the slope of the effect for healthy foods vs. tasty would fall halfway between the analogous slopes in the two exposure conditions (i.e., we assumed that health exposure would shift the slopes towards more preference for healthy foods, whereas hedonic exposure would shift the slopes towards more preference for tasty foods). Thus, in a second power analysis using Monte Carlo simulation (with 10,000 simulations), we assumed that the likely effect size would be half of the effect size observed in the Pilot experiment between the healthy and hedonic exposure groups (an estimated regression coefficient for the exposure X food type interaction of .09). Using the same procedure to assess power just described, we found that a sample size of 80 participants in each condition would have 81% power. Because it's possible that the neutral baseline wouldn't fall exactly half way between the hedonic and health exposure conditions in the planned experiment, we opted for a sample size of 100 participants. This sample size establishes 87% power to detect the two predicted interactions if the estimated regression coefficient were exactly half the size as the original (.09), thereby making it possible to detect a somewhat weaker interaction when comparing the neutral baseline with one of the exposure conditions. On the basis of this analysis, we planned to include 100 participants in each of the 3 conditions for Part A of the main experiment, for a total sample size of 300. Additionally, this sample size had 100% power to detect the original interaction between hedonic and health exposure in the planned experiment (i.e., for the estimated regression coefficient of .18).

As observed in the results for the experiment reported in the article (and also for Parts A and B in the SM), the standardized coefficient for the exposure X food type interaction was smaller than the .18 coefficient in the Pilot experiment. Given these revised estimates of the interaction's effect size, we overestimated power in our original analyses, which explains the patterns of significance for the later exposure X food type interaction in the main experiment.

Images and descriptions for the foods and gifts

Figures SM-1 and SM-2 presents the images and descriptions for the 48 foods. Figure SM-1 presents the 24 tasty foods, organized in columns by eating situations. Figure SM-2 presents the 24 healthy foods.

Figures SM-3 presents the images and descriptions for the 12 birthday gifts used as fillers, including the 6 indulgent gifts and the 12 healthy gifts.

Figure SM-1. Images and descriptions of the tasty foods

BREAKFAST	LUNCH	DINER	SNACK
ROLL WITH BACON 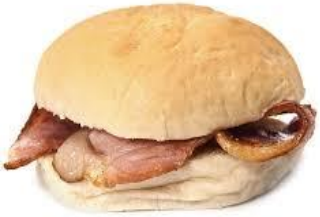	SAUSAGE ROLL 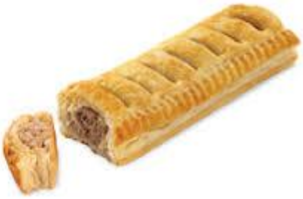	PEPPERONI AND CHEESE PIZZA 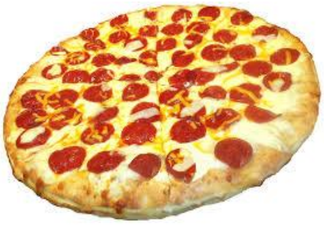	CHOCOLATE CAKE 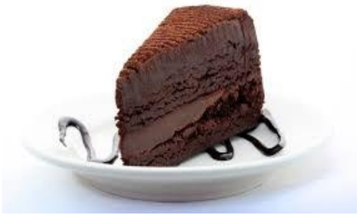
PANCAKES WITH MAPLE SYRUP 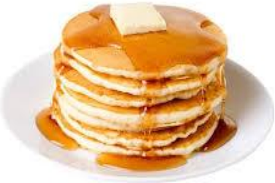	HAM AND CHEESE TOASTIE 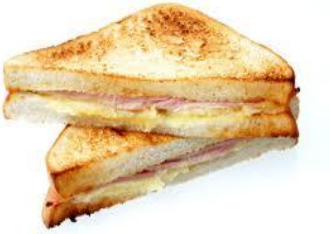	MACARONI AND CHEESE 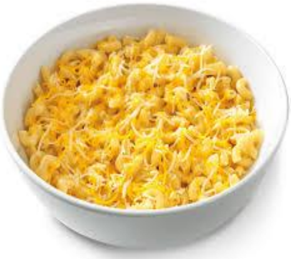	CHOCOLATE CARAMEL DIGESTIVES 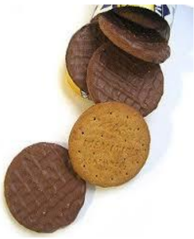
FULL ENGLISH BREAKFAST 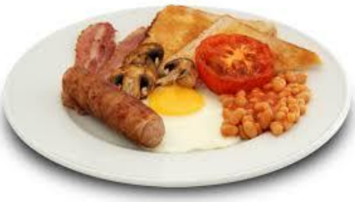	CHIPS AND CHEESE 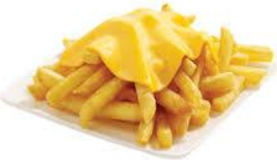	BACON CHEESEBURGER 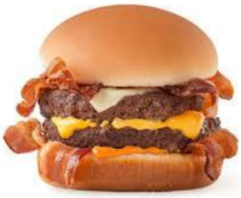	FLAPJACK 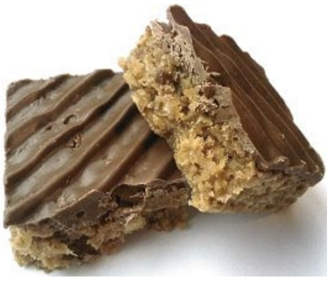
SAUSAGE SANDWICH 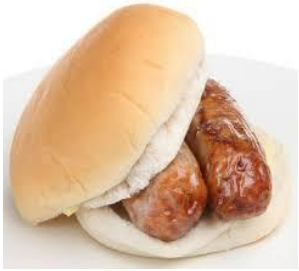	PORK PIE 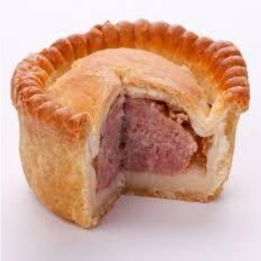	FISH AND CHIPS 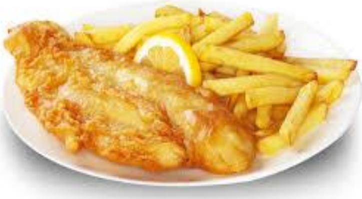	MARS BAR 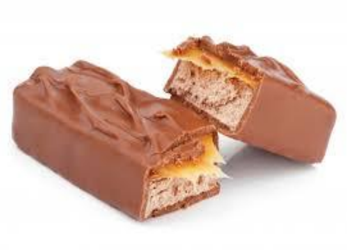
FRENCH TOAST 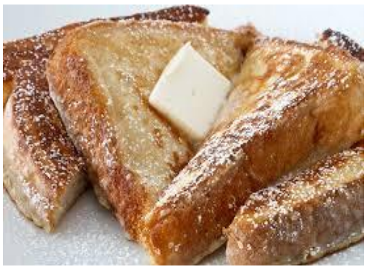	STEAK BAKE 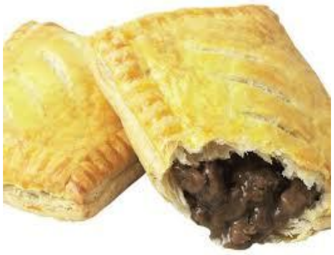	TAKEAWAY LAMB CURRY 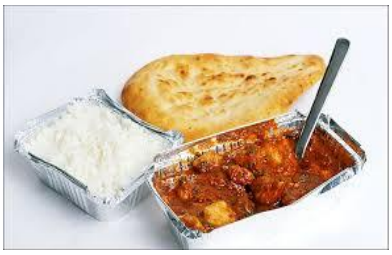	SCONE WITH CLOTTED CREAM AND JAM 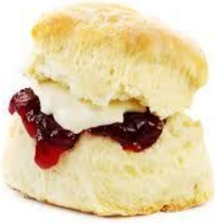
CHOCOLATE CREPE 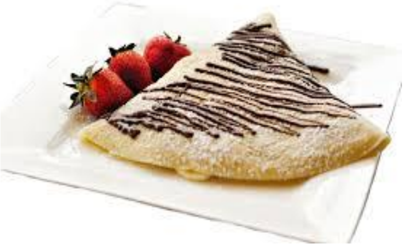	CHILI AND CHEESE BURRITO 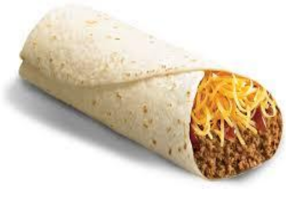	DONER KEBAB 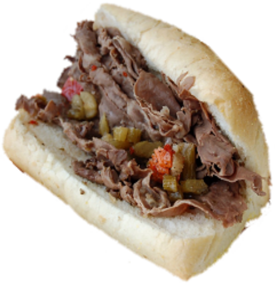	CHEESE AND ONION CRISPS 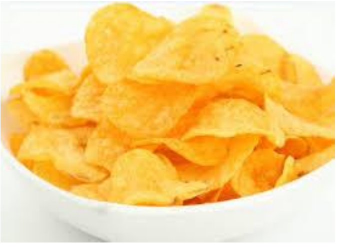

Figure SM-2. Images and descriptions of the healthy foods

BREAKFAST

PORRIDGE WITH MILK

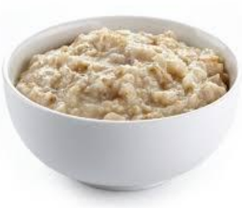

BRANFLAKES WITH BANANA

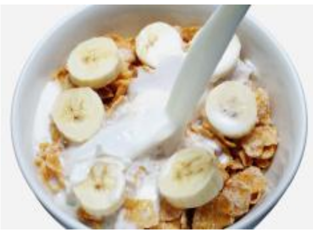

BERRIES WITH YOGURT

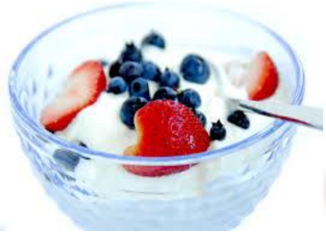

MUESLI AND MILK

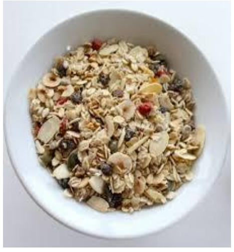

POACHED EGG ON WHOLEMEAL TOAST

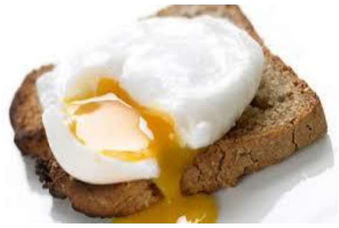

SALMON ON MULTISEED BAGEL

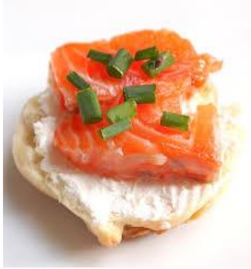

LUNCH

CHICKEN SALAD SANDWICH

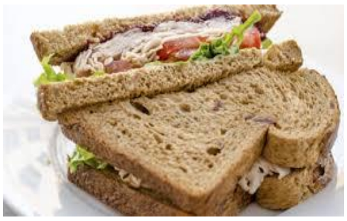

BAKED POTATO

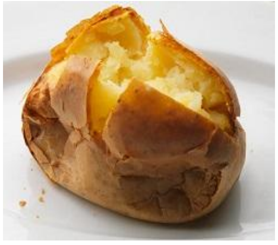

TUNA SALAD SANDWICH

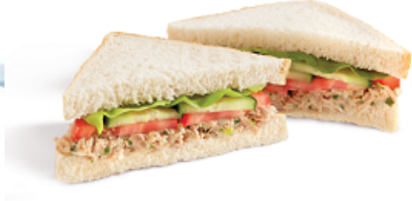

FALAFEL WRAP

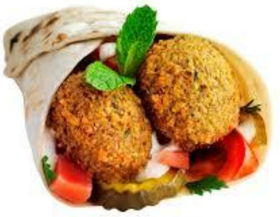

SUPER FOOD SALAD

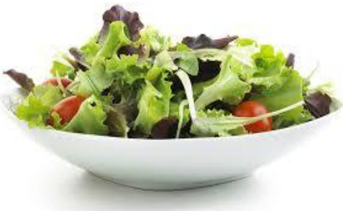

TOMATO SOUP

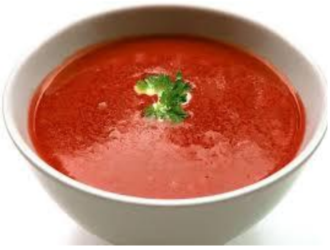

DINER

VEGETABLE STIR FRY

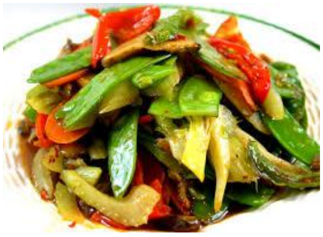

BAKED FISH WITH VEGETABLES

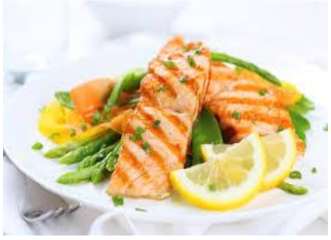

SUSHI

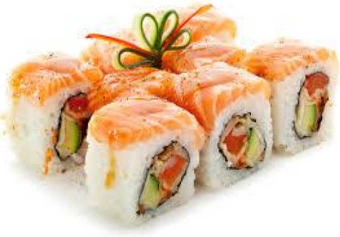

TURKEY AND ROASTED VEGETABLES

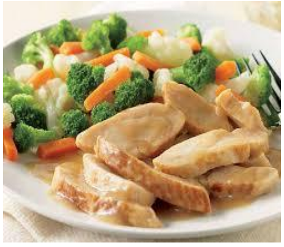

SPINACH QUICHE

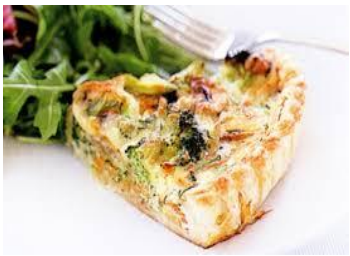

CHICKEN BREAST SALAD

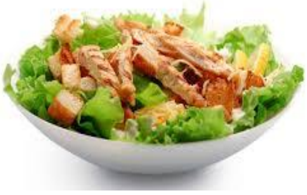

SNACK

VEGETABLES AND HUMMUS

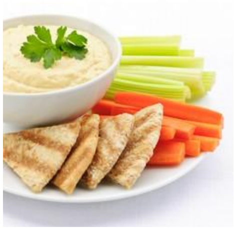

DRIED FRUITS AND NUTS

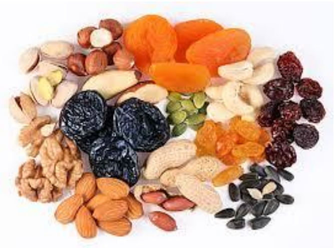

OATCAKES

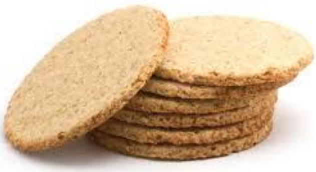

PLAIN YOGURT

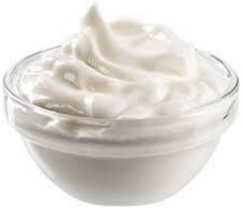

FRESH FRUIT SALAD

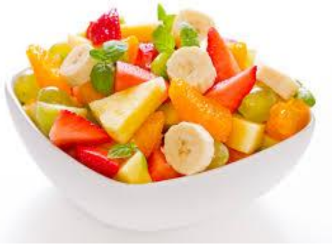

RAW FRUIT AND NUT BAR

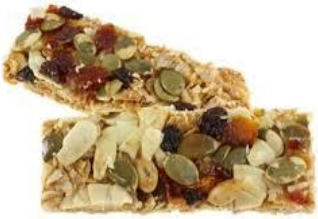

Figure SM-3. Images and descriptions of the birthday gifts

INDULGENT

BOX THEATRE TICKETS

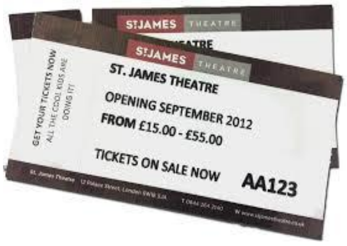

DESIGNER WATCH

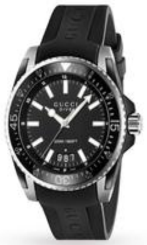

COCKTAIL
MAKING MASTERCLASS

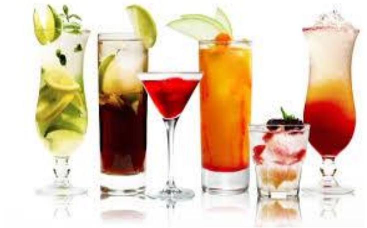

FOUR STAR HOTEL STAY

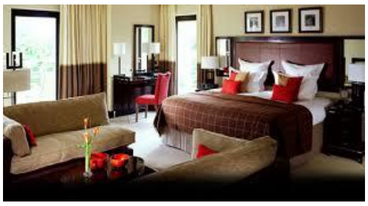

IPOD

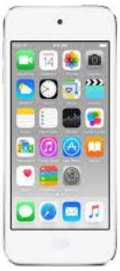

ELECTRIC GUITAR

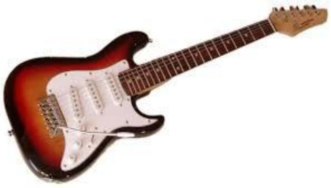

HEALTHY

FITNESS TRACKER
WRISTBAND

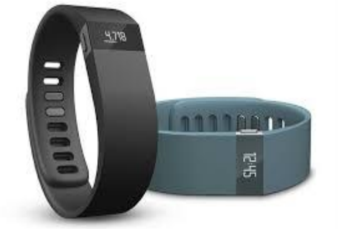

TRAINING SHOES

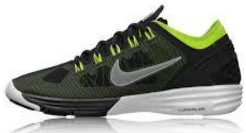

EXERCISE BALL

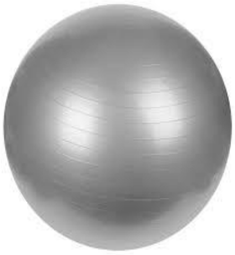

ELECTRIC TOOTHBRUSH

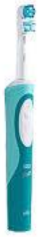

FOOTBALL

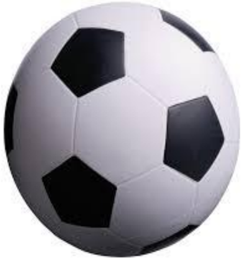

WEIGHTED HULA HOOP

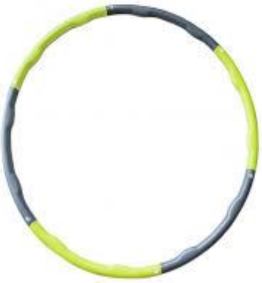

Average endorsement, preference, and frequency for the 48 foods

The following two tables list the tasty foods (Tables SM-1) and the healthy foods (Tables SM-2). For each food, a code is presented in the third column so that the food can be easily identified in the data files made publically available.

In the next two columns, each food's average endorsement in each exposure condition is presented for the hedonic exposure and health exposure groups. The scores are in the original units of the endorsements measure, ranging from +5 to -5 (standardized in all later analyses). As described in the article, this range indicated a high to low number of hedonic features in the hedonic exposure group, and analogously, a high to low number of health features in the health exposure group.

In in the next three columns, each food's average preference judgment is presented for each exposure group (hedonic exposure, no exposure, and health exposure). The scores are in the original units of the food preference measure, ranging from -3 to +3 (standardized in all later analyses). As described in the article, this range indicated a high to low intention to consume a food for a particular meal, with scale labels for: definitely not, probably not, not sure, probably, definitely.

In the final three columns, each food's average consumption frequency judgment is presented for each exposure group. The scores are in the original units of the consumption frequency measure, ranging from 0 to 10 (standardized in all later analyses). As described in the article, this range indicated a high to low frequency of consuming a food at a particular meal, with scale labels for: never, a few times a year, about once a month, a few times a month, about one time a week, a few times a week, typically daily.

Table SM-1. Average endorsement, preference, and frequency for each tasty food as a function of exposure (Hedonic, No Exposure, Health).

Tasty foods			Endorsement		Preference			Frequency		
**Meal**	**Food**	**Code**	**Hedonic**	**Health**	**Hedonic**	**No Exposure**	**Health**	**Hedonic**	**No Exposure**	**Health**
Breakfast	Roll with bacon	f25	0.86	-3.92	0.79	0.98	0.68	2.90	2.74	2.79
Breakfast	Pancakes with maple syrup	f26	3.07	-3.91	1.53	1.40	1.27	2.24	2.07	1.96
Breakfast	Full english breakfast	f27	2.34	-3.5	1.58	1.48	1.46	3.15	2.94	2.97
Breakfast	Sausage sandwich	f28	0.87	-3.9	0.85	0.87	0.73	2.95	2.71	2.72
Breakfast	French toast	f29	2.20	-3.49	1.19	0.81	0.82	1.86	1.50	1.49
Breakfast	Chocolate crepe	f30	3.5	-3.66	1.37	0.84	0.77	1.52	1.33	1.20
Lunch	Sausage roll	f31	0.10	-3.90	0.48	0.60	0.42	2.66	2.68	2.69
Lunch	Ham and cheese toastie	f32	0.18	-3.12	0.99	1.24	1.04	2.08	3.42	1.89
Lunch	Chips and cheese	f33	1.67	-4.29	0.58	0.48	0.24	2.25	2.10	1.97
Lunch	Pork pie	f34	-0.78	-3.97	-0.96	-0.73	-1.08	1.26	1.14	1.11
Lunch	Steak bake	f35	0.27	-3.81	-0.06	0.20	-0.17	1.85	1.89	1.69
Lunch	Chili and cheese burrito	f36	1.93	-3.34	0.58	0.51	0.23	1.54	1.42	1.10
Diner	Pepperoni and cheese pizza	f37	2.04	-4.31	1.42	1.34	1.04	3.98	3.78	3.87
Diner	Macaroni and cheese	f38	0.30	-3.51	0.58	0.79	0.52	2.59	2.59	2.39
Diner	Bacon cheeseburger	f39	2.36	-4.40	0.91	0.92	0.77	2.74	2.68	2.58
Diner	Fish and chips	f40	1.92	-3.63	1.33	1.23	1.12	3.29	3.15	3.00
Diner	Takeaway lamb curry	f41	2.66	-3.52	0.71	0.51	0.39	2.30	1.99	2.11
Diner	Doner kebab	f42	1.77	-4.35	-0.03	-0.13	-0.78	1.69	1.71	1.29
Snack	Chocolate cake	f43	3.57	-4.31	1.53	1.26	1.30	3.42	3.12	3.19
Snack	Chocolate caramel digestives	f44	1.64	-3.82	1.26	1.31	1.32	4.19	3.66	4.01
Snack	Flapjack	f45	1.72	-3.23	1.25	1.32	1.13	2.99	2.79	2.80
Snack	Mars bar	f46	1.68	-4.26	1.10	0.91	0.93	3.45	3.09	3.34
Snack	Scone with clotted cream and jam	f47	2.52	-3.61	1.22	0.85	0.97	2.16	1.94	2.07
Snack	Cheese and onion crisps	f48	0.48	-3.77	1.00	0.79	0.76	4.94	4.21	4.38
		Average	1.62	-3.81	0.88	0.82	0.66	2.67	2.53	2.44

Table SM-2. Average endorsement, preference, and frequency for each healthy food as a function of exposure (Hedonic, No Exposure, Health).

**Healthy foods**			**Endorsement**		**Preference**			**Frequency**		
**Meal**	**Food**	**Code**	**Hedonic**	**Health**	**Hedonic**	**No Exposure**	**Health**	**Hedonic**	**No Exposure**	**Health**
Breakfast	Porridge with milk	f01	-2.91	2.55	0.09	0.78	0.50	4.08	3.94	3.59
Breakfast	Branflakes with banana	f02	-2.03	3.36	0.13	0.37	0.40	2.76	2.50	2.50
Breakfast	Berries with yogurt	f03	-0.62	3.08	0.87	1.05	1.06	3.38	3.31	2.88
Breakfast	Muesli and milk	f04	-2.70	3.02	-0.46	0.11	0.17	2.63	2.59	2.61
Breakfast	Poached egg on wholemeal toast	f05	-0.37	2.23	1.04	1.14	1.08	3.40	3.21	3.28
Breakfast	Salmon on multiseed bagel	f06	1.39	2.52	-0.10	-0.32	-0.39	1.29	1.17	1.18
Lunch	Chicken salad sandwich	f07	-1.10	2.34	0.89	1.08	0.99	3.93	3.55	3.85
Lunch	Baked potato	f08	-1.70	1.22	0.79	1.09	1.01	3.44	3.31	3.25
Lunch	Tuna salad sandwich	f09	-1.65	2.51	-0.14	0.10	0.27	2.82	2.87	3.09
Lunch	Falafel wrap	f10	0.73	1.25	0.53	0.66	0.45	1.83	1.88	1.77
Lunch	Super food salad	f11	-2.21	4.36	0.11	0.17	0.45	3.37	3.18	3.33
Lunch	Tomato soup	f12	-1.51	3.01	0.56	0.86	0.71	3.19	3.12	2.97
Diner	Vegetable stir fry	f13	-0.53	3.54	0.91	1.32	1.21	3.88	3.61	3.87
Diner	Baked fish with vegetables	f14	-0.23	3.92	0.69	0.72	0.75	3.37	3.12	3.12
Diner	Sushi	f15	1.51	2.75	-0.17	-0.24	-0.30	1.68	1.63	1.41
Diner	Turkey and roasted vegetables	f16	-0.58	3.06	0.79	1.12	1.09	2.96	2.89	3.03
Diner	Spinach quiche	f17	-0.46	1.16	-0.23	0.14	0.16	1.74	1.46	1.81
Diner	Chicken breast salad	f18	-1.13	3.77	0.68	0.81	1.07	3.31	3.00	3.29
Snack	Vegetables and hummus	f19	-0.49	3.38	0.37	0.50	0.60	3.15	3.11	2.96
Snack	Dried fruits and nuts	f20	-1.53	2.74	-0.08	0.61	0.35	3.39	3.45	3.74
Snack	Oatcakes	f21	-2.51	2.09	-0.93	-0.45	-0.40	1.58	1.53	1.83
Snack	Plain yogurt	f22	-2.25	2.00	-0.12	-0.11	-0.12	3.21	2.93	2.96
Snack	Fresh fruit salad	f23	-0.21	3.97	1.32	1.44	1.38	4.28	4.34	4.25
Snack	Raw fruit and nut bar	f24	-1.90	2.44	-0.64	-0.15	-0.06	2.33	2.40	2.52
		Average	-1.04	2.76	0.29	0.53	0.52	2.96	2.84	2.88

Tables and figures for Experiment Parts A and B

This section of the SM presents the individual results for Parts A and B of the combined experiment reported in the article.

The figures for Parts A and B of the experiment are presented first, followed by the supporting tables.

Figures SOM-4, SOM-5, SOM-6, SOM-7, SOM-8, and SOM-9 here correspond to Figures 2, 3, 4, 5, 6, and 7, in the main text.

Tables SM-3, SM-4, SM-5, SM-6, and SM-7 here correspond to Tables 1, 2, 3, 4, and 5 in the main text.

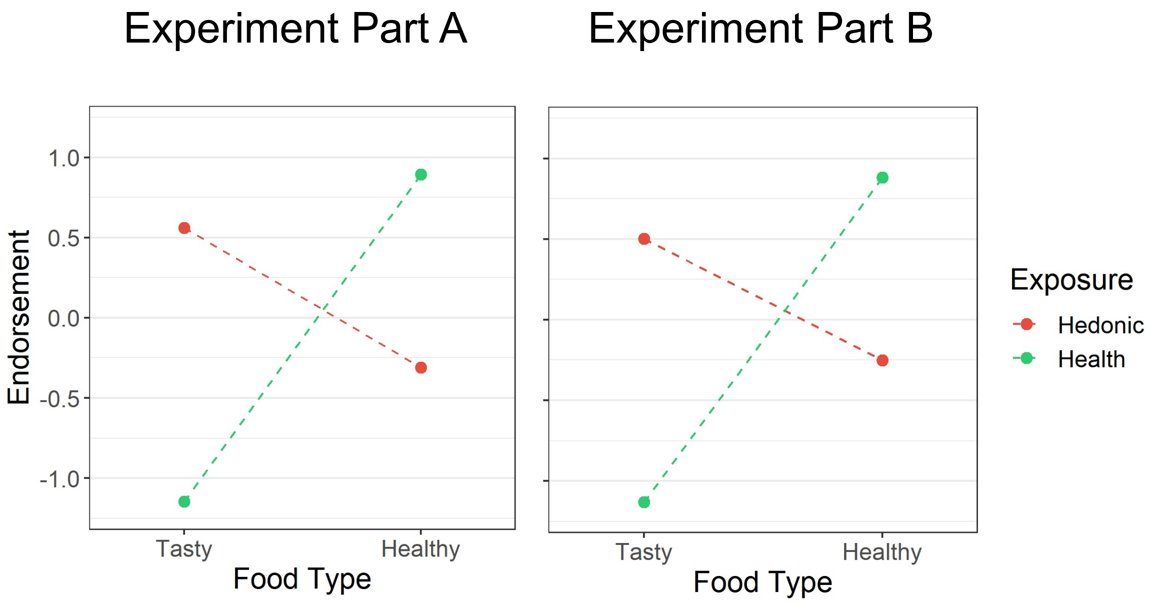

Figure SM-4. Evidence for the validity of the food type manipulation between tasty and healthy foods (for Parts A and B of the experiment reported in the article). Whereas tasty foods were high on hedonic endorsements and low on healthy endorsements, healthy foods were low on hedonic endorsements and high on healthy endorsements. Hedonic endorsements were produced in the hedonic exposure condition, and healthy endorsements were produced in the health exposure condition. A modeled interaction from regression is shown, with the endorsement scale plotted in standardized units.

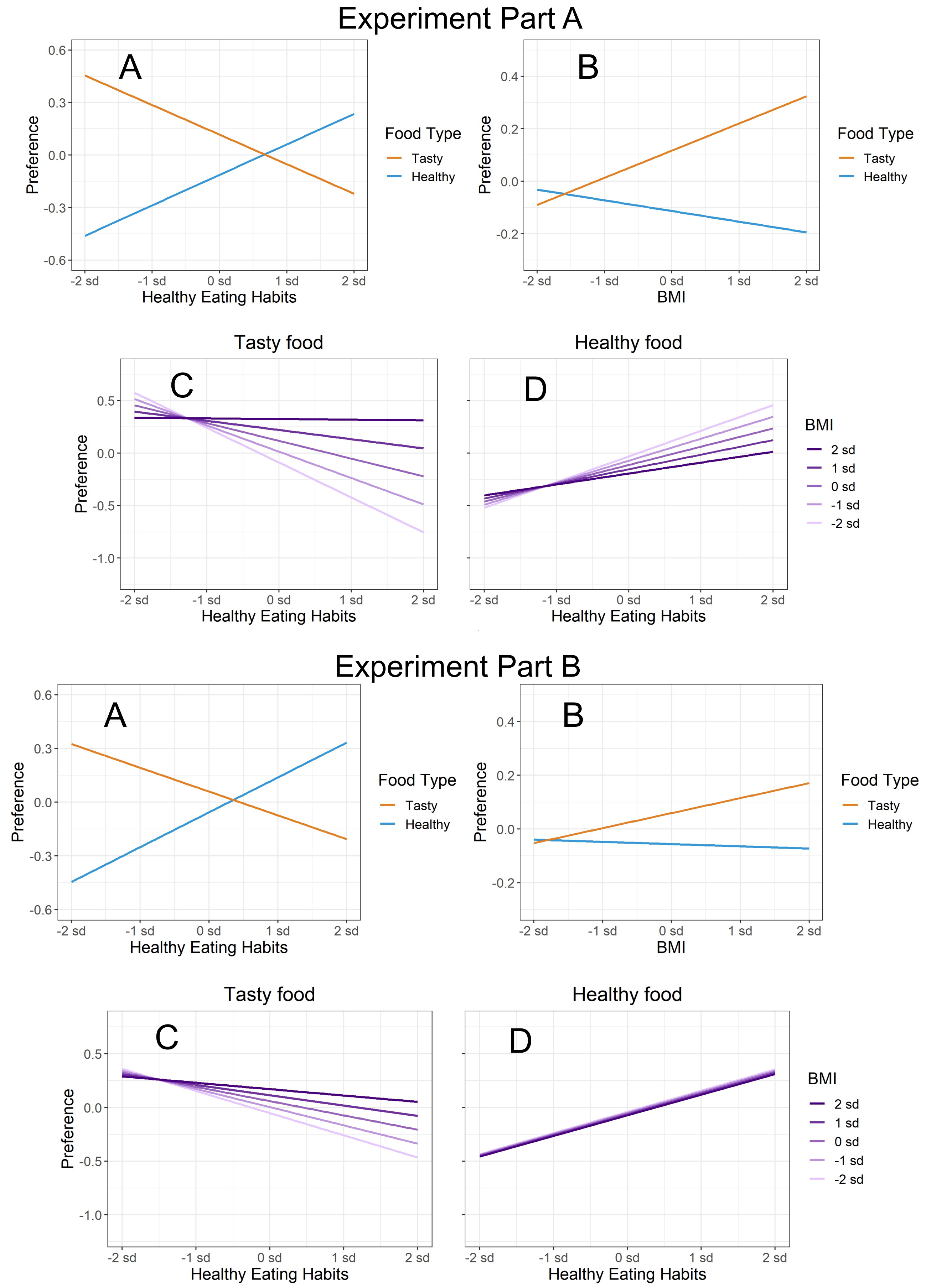

Figure SM-5. Evidence for the validity of the food preference measure (for Parts A and B of the experiment reported in the article). (A) As participants' eating habits become increasingly healthy, preferences for healthy foods increased, whereas preferences for tasty foods decreased. (B) Conversely, as participants' BMI increased, preferences for healthy foods decreased, whereas preferences for tasty foods increased. (C and D) Finally, the food type × eating habits × BMI interaction further shows that BMI modulated the food type × eating habits interaction in panel A. In all panels, a modeled interaction from regression is shown, with all scales plotted in standardized units.

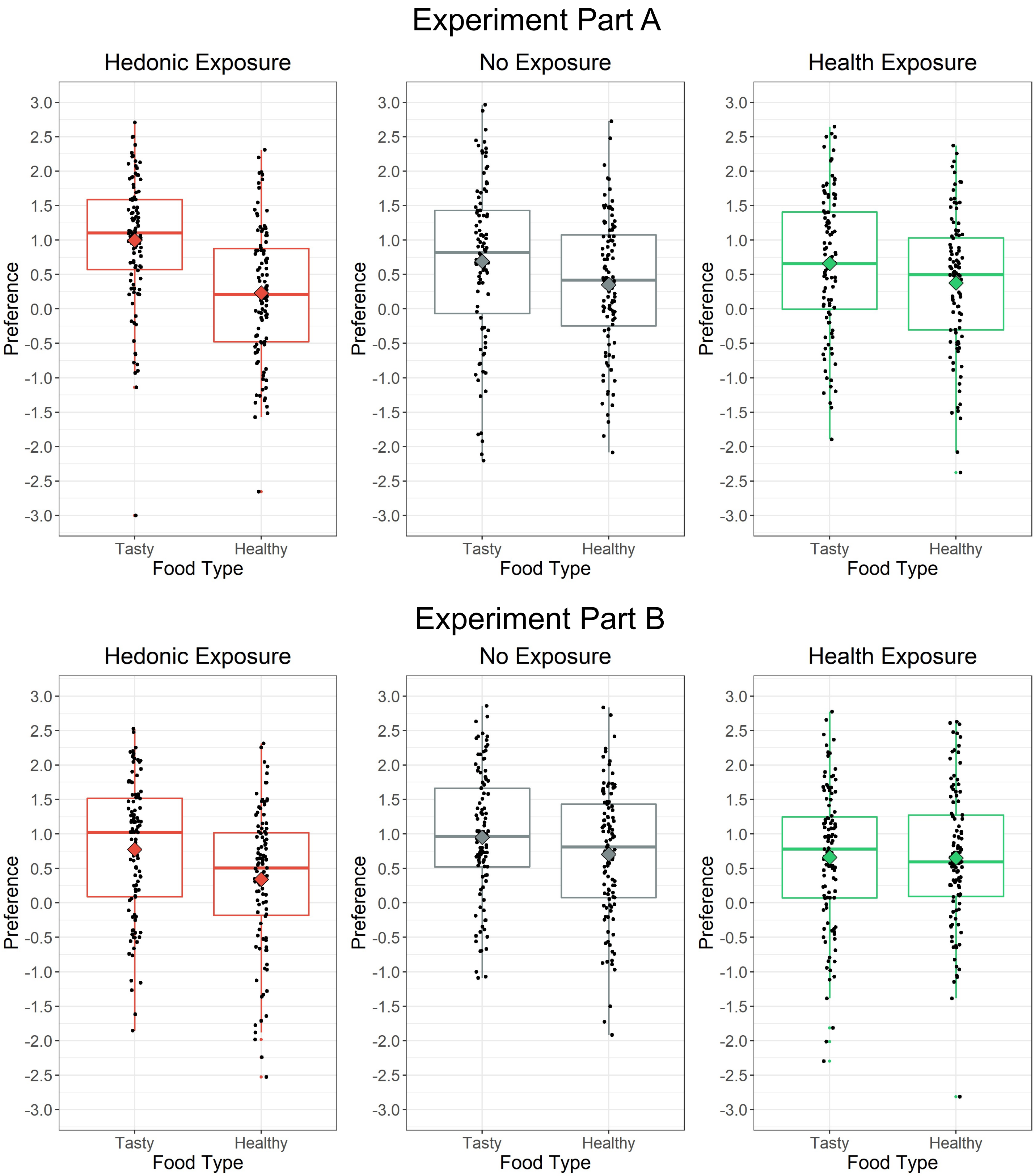

Figure SM-6. Results for the food preference task (for Parts A and B of the experiment reported in the article). The vertical axis represents responses on the original -3 to +3 preference scale. For each exposure group, a diamond represents the mean; a box and whisker plot represents the median and inter-quartile range. Each point represents a participant's average judgment for either the 24 tasty foods or for the 24 healthy foods.

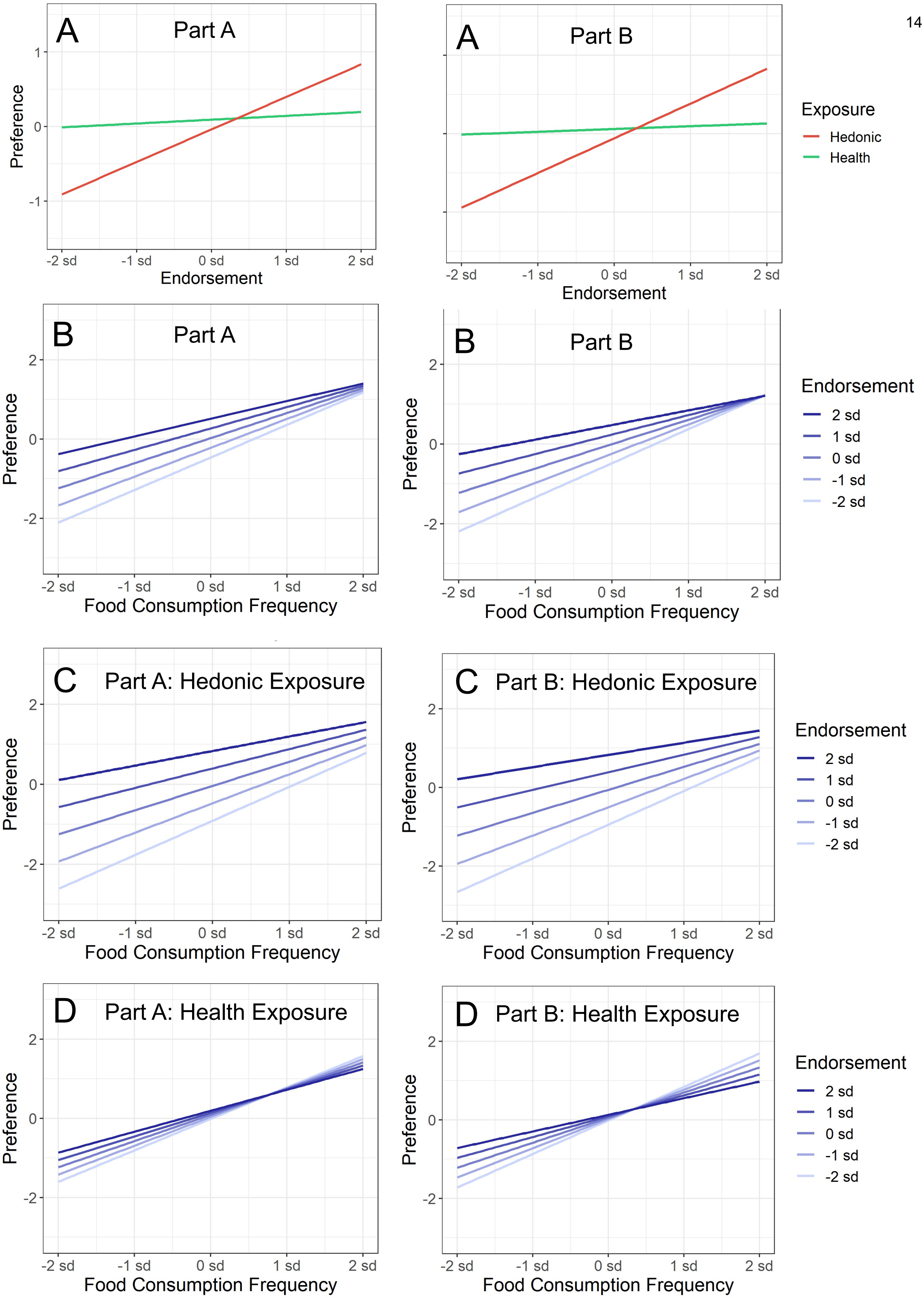

Figure SM-7. For Parts A and B of the experiment reported in the article, Panel A presents the interaction between exposure condition and endorsement on food preference (for increasing hedonic endorsements in the hedonic exposure condition and for increasing healthy endorsements in the health exposure condition). Panel B presents the consumption frequency × endorsement interaction on food preference. Panels C and D present the endorsement × frequency × exposure interaction on food preference for the hedonic and health exposure conditions individually. In each panel, a modeled interaction from regression is shown, with all scales plotted in standardized units.

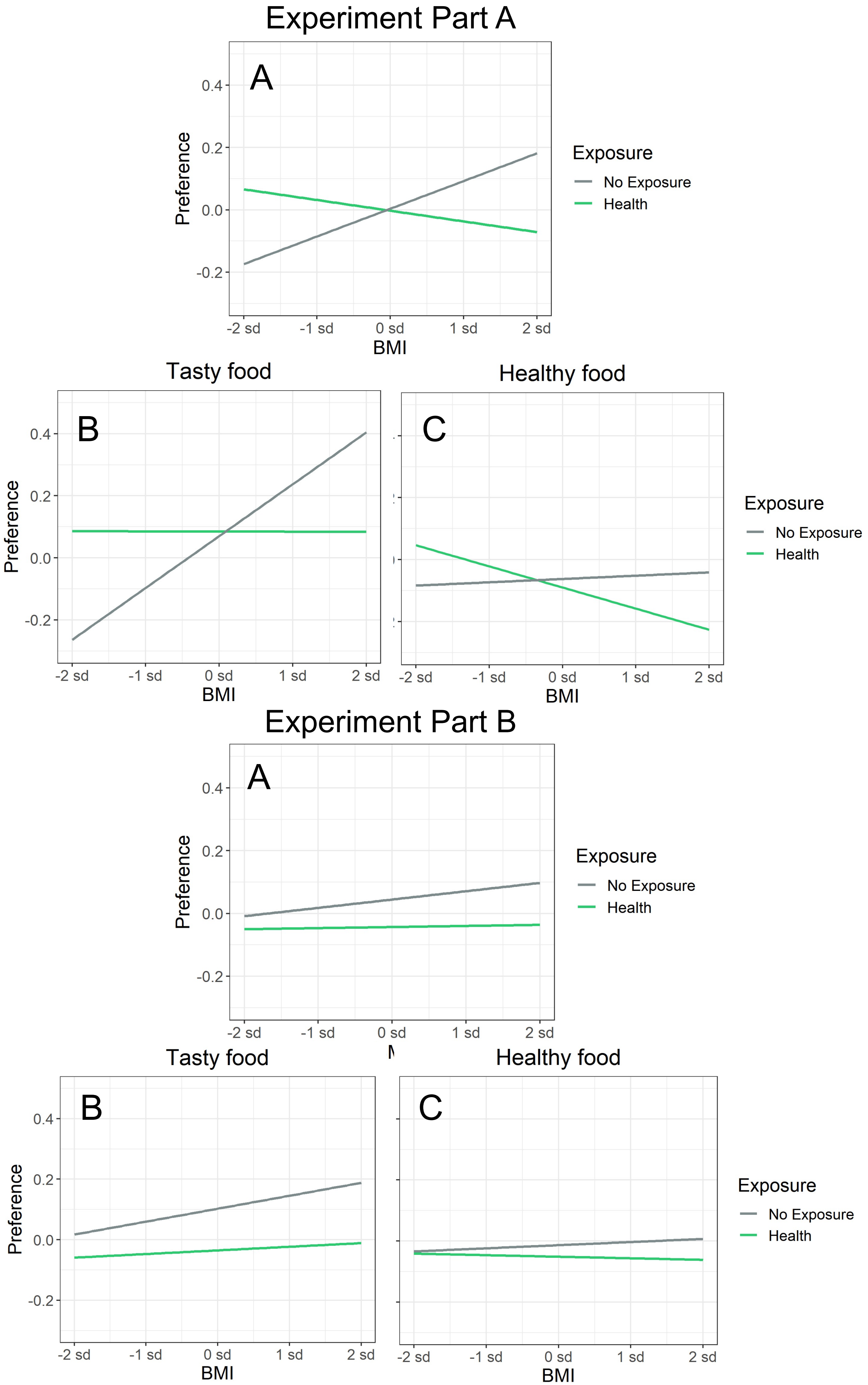

Figure SM-8. For Parts A and B of the experiment reported in the article, Panel A presents the exposure × BMI interaction on food preference. Panels B and C present this interaction individually for tasty and healthy foods, respectively (illustrating the *lack* of an exposure x BMI x food type interaction). In each panel, a modeled interaction from regression is shown, with all scales plotted in standardized units.

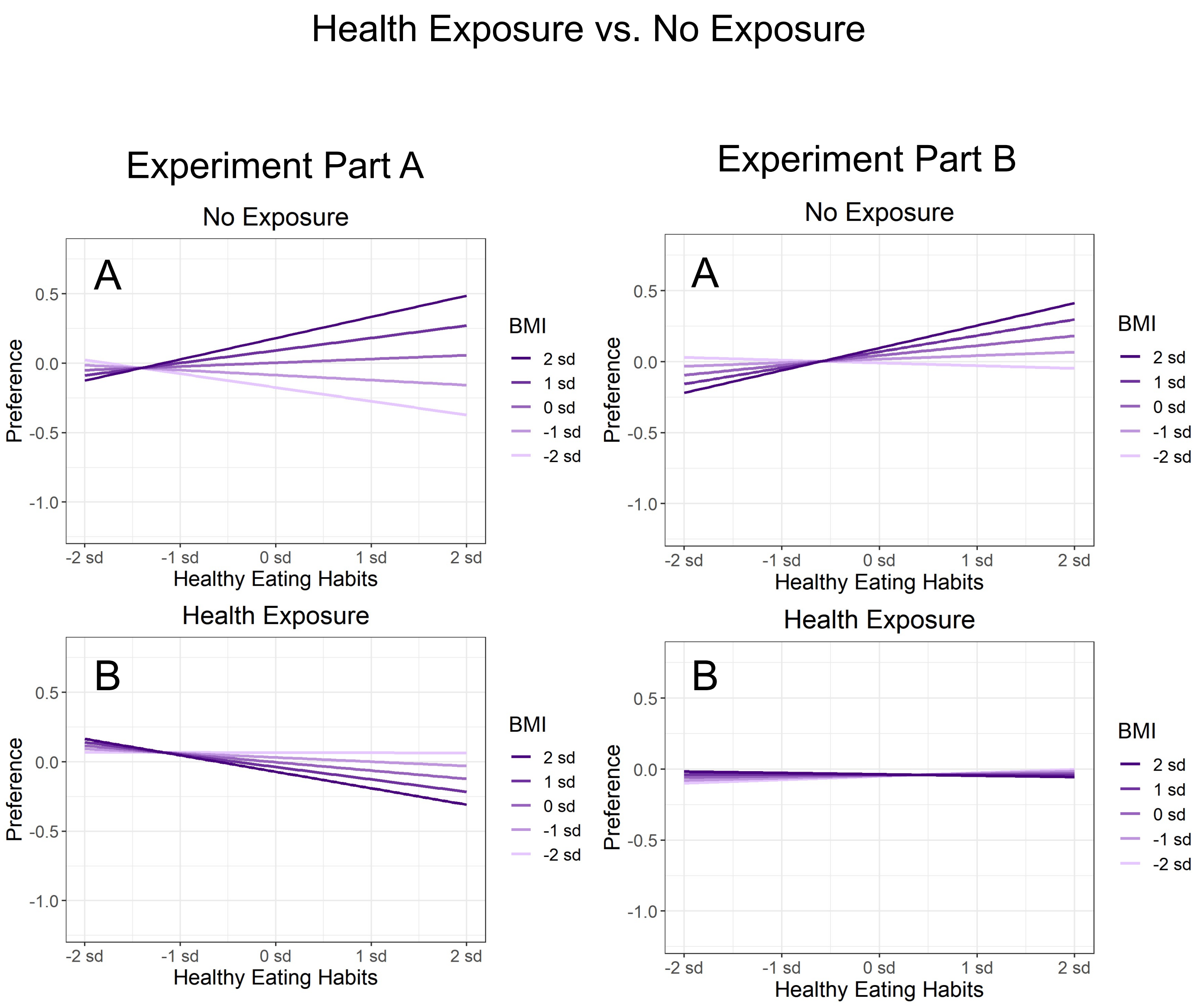

Figure SM-9. For Parts A and B of the experiment reported in the article, Panels A and B present the exposure × healthy eating habits × BMI interaction on food preference for the no-exposure and health exposure groups, respectively (combined across tasty and healthy foods). In each panel, a modeled interaction from regression is shown, with all scales plotted in standardized units.

Table SM-3. Mixed-effect regressions of endorsement on food type and exposure for Parts A and B of the experiment reported in the article.

**DV: Endorsement**	**Model 1**			**Model 2**					**Model 3**	
**Part / Predictor**	**Estimate**	**SE**	***t***	**Estimate**	**SE**	***t***	**R^2**	**AIC**	**ΔR^2**	**AIC**
**Experiment Part A**										
Food Type	-.29	.029	-1.16	-.29	.032	-9.06	75	15289	-4	16383
Exposure	.13	.014	8.97	.13	.032	3.95	74	15120	-4	16396
Food Type x Exposure	.73	.006	131.10	.73	.033	22.31	79	13831	-63	26120
**Experiment Part B**										
Food Type	-.32	.028	-11.23	-.32	.032	-9.73	72	16884	-6	18223
Exposure	.13	.014	8.98	.13	.034	3.71	71	16878	-5	18229
Food Type x Exposure	.69	.006	119.76	.69	.035	19.71	77	15279	-59	27003

Note. Regressions were performed on standardized measures. For Food Type, tasty foods were coded +1, and healthy foods were coded -1. For Exposure, hedonic exposure was coded +1, and health exposure was coded -1.

Table SM-4. Mixed-effect regression of food preference on predictors for food type (Food), healthy eating habits (Habits), and BMI for Parts A and B of the experiment reported in the article. Exposure was not included as a factor, given that the interactions of interest below remained constant across the three exposure conditions (i.e., the regression was performed on all 617 participants).

	**Model 1**			**Model 2**					**Model 3**	
**Part / Predictor**	**Estimate**	**SE**	**t**	**Estimate**	**SE**	**t**	**R2**	**AIC**	**ΔR2**	**AIC**
**Experiment Part A**										
Food Type	.12	.040	2.85	.12	.043	2.67	29	37507	-7	38275
Healthy eating habits	.00	.019	0.13							
BMI	.03	.020	1.59							
Food x Habits	-.17	.007	-23.30	-.17	.021	-8.07	30	37404	-11	38801
Food x BMI	.07	.008	9.58	.07	.020	3.7	29	37458	-8	38359
Food x Habits x BMI	.06	.008	7.15	.06	.019	3.00	31	37312	-9	38318
**Experiment Part B**										
Food type	.06	.037	1.56							
Healthy eating habits	.03	.020	1.52							
BMI	.02	.022	1.08							
Food x Habits	-.16	.007	-22.50	-.16	.019	-8.47	28	39327	-10	40685
Food x BMI	.03	.008	4.08	.03	.019	1.73	28	39370		
Food x Habits x BMI	.02	.008	2.52	.02	.017	1.10	29	39312		

Note. All regressions were performed on standardized measures. Thus, an Estimate is the estimate of a standardized regression coefficient in the respective model, with SE and t being the standard error and t value of the estimate. R2 is the total variance explained by Model 2, and ΔR2 is the amount of variance explained by the main effect or interaction dropped in Model 3. AIC is the value of the Akaike Information Criterion for Models 2 and 3.

Table SM-5. Mixed-effect regressions of food preference on food type (Food) and exposure (Expo) for Parts A and B of the experiment reported in the article.

**DV: food preference**	**Model 1**			**Model 2**					**Model 3**	
**Contrast/Part/Predictor**	**Estimate**	**SE**	***t***	**Estimate**	**SE**	***t***	**R^2**	**AIC**	**ΔR^2**	**AIC**
**Hedonic vs. Health Exposure**										
**Experiment Part A**										
Food Type	.13	.043	2.94	.13	.048	2.61	29	25158	-10	25983
Exposure	.02	.023	0.99							
Food x Expo	.06	.009	6.28	.06	.024	2.40	29	25156	-10	26015
**Experiment Part B**										
Food Type	.05	.038	1.43							
Exposure	-.03	.024	-1.03							
Food x Expo	.05	.009	5.74	.05	.025	2.07	29	26263	-12	27283
**Hedonic vs. No Exposure**										
**Experiment Part A**										
Food Type	.13	.040	3.27	.13	.046	2.83	29	25180	-11	26118
Exposure	.02	.023	0.94							
Food x Expo	.05	.009	5.43	.05	.025	1.98	29	25177	-11	26138
**Experiment Part B**										
Food Type	.08	.037	2.24	.08	.043	1.95	28	26095		
Exposure	-.07	.025	-2.69	-.07	.025	-2.64	19	26846	0	26851
Food x Expo	.02	.009	2.55	.02	.023	0.99	28	26090		
**Health vs. No Exposure**										
**Experiment Part A**										
Food Type	.07	.040	1.86							
Exposure	-.00	.025	-0.04							
Food x Expo	-.01	.009	-0.72							
**Experiment Part B**										
Food Type	.03	.037	0.84							
Exposure	-.04	.025	-1.68							
Food x Expo	-.03	.009	-3.26	-.03	.022	-1.34	27	26684		

Note. Regressions were performed on standardized measures. For Food Type, tasty foods were coded +1, and healthy foods were coded -1. In the Hedonic vs. Health Exposure regression, hedonic exposure was coded +1, and health exposure was coded -1. In the Hedonic vs. No Exposure regression, hedonic exposure was coded +1, and no exposure was coded -1. In the Health vs. No Exposure regression, health exposure was coded +1, and no exposure was coded -1.

Table SM-6. Mixed effect regressions of food preference on predictors that included frequency (Freq) and endorsement (Endo), along with food type (Food) and exposure (Expo), in Parts A and B of the experiment reported in the main article.

DV: food preference	Model 1			Model 2					Model 3	
**Part / Predictor**	**Estimate**	**SE**	***t***	**Estimate**	**SE**	***t***	**R^2**	**AIC**	**ΔR^2**	**AIC**
**Experiment Part A**										
Frequency	.63	.015	41.60	.64	.028	22.70	64	19483	-15	21569
Endorsement	.24	.013	18.05	.26	.022	11.55	62	19571	-3	20301
Food Type	.09	.029	3.24	.10	.032	2.99	60	19637	-2	19991
Exposure	-.07	.025	-2.57	-.07	.027	-2.42	60	19928	-1	19988
Freq x Endo	-.09	.013	-6.98	-.14	.018	-8.17	66	18958	-8	20030
Endo x Expo	.19	.014	14.18	.24	.021	11.18	62	19547	-4	20180
Food x Expo	-.07	.014	-5.18	-.08	.022	-3.77	61	19579	-2	20008
Freq x Endo x Expo	-.03	.014	-2.03	-.03	.016	-1.99	67	18946	-8	19986
Freq x Food x Expo	.05	.015	3.01	.08	.018	4.46	66	18962	-7	19991
Endo x Food x Expo	-.05	.014	-3.56	-.04	.016	-2.69	62	19455	-4	19994
**Experiment Part B**										
Frequency	.61	.014	44.90	.64	.028	22.86	65	20524	-19	23190
Endorsement	.24	.013	18.75	.27	.021	13.31	60	20996	-4	21702
Food Type	.04	.026	1.61							
Exposure	-.06	.025	-2.36	-.06	.026	-2.17	57	21344	0	21363
Freq x Endo	-.12	.012	-9.89	-.16	.016	-10.57	67	20094	-11	21455
Freq x Expo	-.03	.013	-2.07	-.02	.021	-0.88	65	20503		
Endo x Expo	.20	.013	15.86	.22	.021	10.78	60	20989	-4	21606
Food x Expo	-.07	.013	-5.42	-.10	.020	-4.88	59	20921	-2	21387
Freq x Endo x Food	-.03	.012	-2.14	-.03	.015	-1.83	68	19918		
Freq x Food x Expo	.08	.013	5.93	.07	.014	5.09	67	20084	-10	21393

Note. Regressions were performed on standardized measures. For Food Type, tasty foods were coded +1, and healthy foods were coded -1. For Exposure, hedonic exposure was coded +1, and health exposure was coded -1.

Table SM-7. Mixed-effect regressions of food preference on predictors that included healthy habits (Habits) and BMI, along with food type (Food) and exposure (Expo) for Parts A and B of the experiment reported in the main article.

DV: food preference	Model 1			Model 2					Model 3	
Contrast/Part/Predictor	Estimate	SE	*t*	Estimate	SE	*t*	R^2	AIC	ΔR^2	AIC
**Hedonic vs. No Exposure**										
**Experiment Part A**										
Food Type	.14	.040	3.44	.14	.044	3.15	29	25088	-6	25544
Exposure	.02	.022	0.97							
Healthy eating habits	.04	.023	1.58							
BMI	.06	.024	2.39	.06	.026	2.21	23	25517	1	25539
Food x Expo	.06	.009	6.26	.06	.020	2.81	29	25086	-7	25573
Food x Habits	-.18	.009	-19.93	-.18	.025	-7.23	30	25000	-11	25923
Food x BMI	.10	.010	9.79	.10	.023	4.08	30	25063	-7	25629
Habits x BMI	.06	.027	2.26	.06	.028	2.20	25	25413	-2	25539
Food x Expo x Habits	.05	.009	5.90	.05	.020	2.70	31	25001	-8	25569
Food x Expo x BMI	.02	.010	2.01	.02	.021	0.92	30	25066		
Food x Habits x BMI	.08	.011	7.43	.08	.024	3.33	31	24947	-8	25589
**Experiment Part B**										
Food Type	.09	.037	2.32	.09	.041	2.10	28	26052	-6	26545
Expo	-.07	.025	-2.77	-.07	.025	-2.71	21	26543	0	26548
Healthy eating habits	.05	.025	1.90							
BMI	.03	.027	1.04							
Food x Expo	.03	.009	3.60	.03	.021	1.58	28	26049		
Food x Habits	-.14	.009	-15.99	-.14	.023	-6.28	29	26018	-9	26792
Food x BMI	.05	.010	5.43	.05	.022	2.45	28	26049	-7	26569
Food x Expo x BMI	.04	.010	3.79	.04	.022	1.71	28	26051		
**Health vs. No Exposure**										
**Experiment Part A**										
Food Type	.08	.040	1.94							
Expo	-.00	.024	-0.13							
Healthy eating habits	-.02	.024	-0.68							
BMI	.03	.026	1.03							
Food x Habits	-.19	.009	-2.67	-.19	.023	-8.22	29	24872	-10	25735
Food x BMI	.06	.010	5.69	.06	.024	2.34	29	24874	-7	25350
Expo x BMI	-.06	.026	-2.33	-.06	.026	-2.33	24	25299	-1	25323
Food x Expo x Habits	.03	.009	3.32	.03	.019	1.55	30	24875		
Food x Expo x BMI	-.02	.010	-2.25	-.02	.021	-1.07	29	24887		
Food x Habits x BMI	.06	.010	5.93	.06	.023	2.63	30	24803	-8	25353
**Experiment Part B**										
Food Type	.03	.037	0.89							
Expo	-.04	.025	-1.76							
Healthy eating habits	.04	.025	1.53							
BMI	.01	.028	0.53							
Food x Expo	-.02	.009	-2.83	-.03	.018	-1.36	27	26630		
Food x Habits	-.16	.009	-18.34	-.16	.021	-7.75	28	26601	-9	27372
Food x Expo x Habits	-.03	.009	-3.07	-.03	.019	-1.46	28	26605		
Food x Habits x BMI	.02	.009	2.27	.02	.020	1.05	28	26593		

Note. Regressions were performed on standardized measures. For Food Type, tasty foods were coded +1, and healthy foods were coded -1. In the Hedonic vs. No Exposure regression, hedonic exposure was coded +1, and no exposure was coded -1. In the Health vs. No Exposure regression, health exposure was coded +1, and no exposure was coded -1.
